# Structures of atypical chemokine receptor 3 reveal the basis for its promiscuity and signaling bias

**DOI:** 10.1126/sciadv.abn8063

**Published:** 2022-07-13

**Authors:** Yu-Chen Yen, Christopher T. Schafer, Martin Gustavsson, Stefanie A. Eberle, Pawel K. Dominik, Dawid Deneka, Penglie Zhang, Thomas J. Schall, Anthony A. Kossiakoff, John J. G. Tesmer, Tracy M. Handel

**Affiliations:** ^1^ Department of Biological Sciences, Purdue University, West Lafayette, IN, USA.; ^2^ Skaggs School of Pharmacy and Pharmaceutical Sciences, University of California, San Diego, La Jolla, CA, USA.; ^3^ Department of Biomedical Sciences, Faculty of Health and Medical Sciences, University of Copenhagen, 2200 Copenhagen, Denmark.; ^4^ Department of Biochemistry and Molecular Biology, University of Chicago, Chicago, IL, USA.; ^5^ Department of Biophysics, Jagiellonian University, Krakow, Poland.; ^6^ ChemoCentryx Inc., 835 Industrial Rd., Suite 600, San Carlos, CA 94070, USA.; ^7^ Department of Molecular Pharmacology and Medicinal Chemistry, Purdue University, West Lafayette, IN, USA.

## Abstract

Both CXC chemokine receptor 4 (CXCR4) and atypical chemokine receptor 3 (ACKR3) are activated by the chemokine CXCL12 yet evoke distinct cellular responses. CXCR4 is a canonical G protein–coupled receptor (GPCR), whereas ACKR3 is intrinsically biased for arrestin. The molecular basis for this difference is not understood. Here, we describe cryo-EM structures of ACKR3 in complex with CXCL12, a more potent CXCL12 variant, and a small-molecule agonist. The bound chemokines adopt an unexpected pose relative to those established for CXCR4 and observed in other receptor-chemokine complexes. Along with functional studies, these structures provide insight into the ligand-binding promiscuity of ACKR3, why it fails to couple to G proteins, and its bias toward β-arrestin. The results lay the groundwork for understanding the physiological interplay of ACKR3 with other GPCRs.

## Teaser

Structures of an atypical chemokine receptor show a novel ligand pose and shed light on its plasticity and arrestin bias.

## INTRODUCTION

The chemokine CXCL12 and two of its receptors, CXC chemokine receptor 4 (CXCR4) and atypical chemokine receptor 3 (ACKR3), together play critical roles in central nervous system, cardiac, and vascular development, as well as in cancers where they contribute to angiogenesis, tumor growth, invasion, and metastasis (*1*, *2*). CXCR4 is a canonical G protein (heterotrimeric guanine nucleotide–binding protein)–coupled receptor (GPCR) that regulates cell movement in response to CXCL12. In contrast, ACKR3 does not activate G proteins and is intrinsically β-arrestin biased (*3*, *4*). One of the best described functions of ACKR3 is “scavenging,” whereby it regulates extracellular concentrations of CXCL12 to shape chemokine gradients and maintain CXCR4 responsiveness by preventing overstimulation and down-regulation of the receptor (*5*, *6*). Scavenging occurs by constitutive internalization and recycling of ACKR3 and concomitant transport of CXCL12 to lysosomes for degradation (*7*). The importance of this function has been demonstrated in vivo, where the absence of ACKR3 scavenging leads to profound defects in CXCR4-mediated migration of cortical interneurons in mice (*5*). Scavenging of CXCL12 by ACKR3 has also been shown to promote growth and metastasis of CXCR4^+^ breast cancer cells and is thought to contribute to the progression of other cancers (*2*). The importance of scavenging is further underscored by the recent discovery of GPR182, another atypical receptor that promotes hematopoietic stem cell maintenance and retention in the bone marrow by scavenging CXCL12 and likely influencing CXCR4 (*8*).

ACKR3 scavenges a diverse array of other ligands including chemokines (e.g., CXCL11, the viral chemokine vCCL20, and CXCL12 variants), nonchemokine proteins (e.g., adrenomedullin and MIF), and peptides (e.g., pro-enkephalin–derived BAM22 and opioids) (*9*, *10*), suggesting that it could serve as a pleiotropic regulator of many other GPCRs in various biological contexts. How it recognizes and scavenges these disparate ligands is not understood. It is also unclear why most ligands are activating with respect to arrestin recruitment (*11*), suggesting that ACKR3 is readily activated, unlike other chemokine receptors, which are readily antagonized. The molecular basis for its bias toward β-arrestins over heterotrimeric G proteins, which contrasts with closely related canonical chemokine receptors, is particularly puzzling. To address these questions, we used cryo–electron microscopy (cryo-EM) single-particle analysis to determine multiple unique structures of ACKR3 bound to a series of agonists: wild-type CXCL12 (CXCL12_WT_), CXCL12_LRHQ_ (a higher-affinity variant of CXCL12 with alterations at its N terminus) (*12*), and the small-molecule agonist CCX662 (*13*, *14*). Our structural and functional data provide insights into the promiscuous recognition of ligands by ACKR3 and the molecular determinants that control its unique pharmacological behavior relative to canonical chemokine receptors.

## RESULTS

**Fig. 1.**
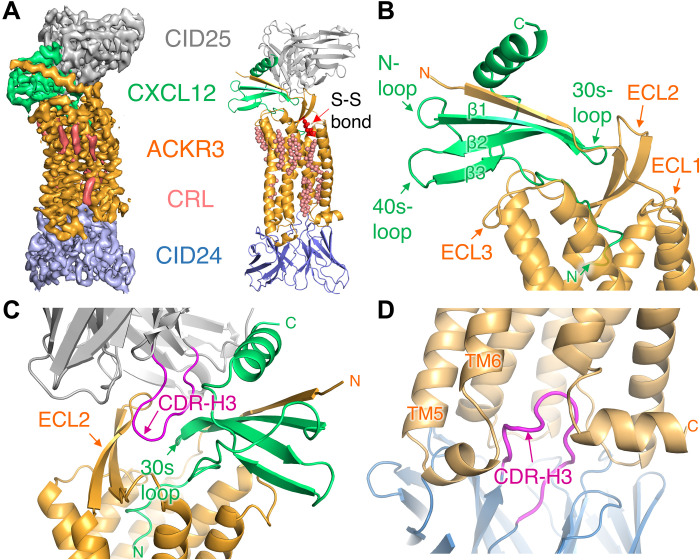
Overview of the CID25-ACKR3·CXCL12-CID24 complex (PDB entry 7SK3). (**A**) Cryo-EM map contoured at 10 σ (left) and corresponding atomic model (right). CRL, cholesterol. (**B**) Interactions of ACKR3 (orange cartoon) with CXCL12 (green cartoon). The N terminus of the receptor contributes an extra strand to the core β sheet of the chemokine, whereas the N terminus of CXCL12 extends into the orthosteric pocket of the receptor. (**C**) CDR-H3 of the heavy chain of CID25 (gray cartoon with magenta CDR-H3) interacts with ECL2 of the receptor (orange cartoon) and the 30s loop of chemokine (shown in green cartoon). (**D**) CDR-H3 of the heavy chain of CID24 (blue cartoon with magenta CDR-H3) inserts into the cytoplasmic pocket of the active receptor.

To facilitate structure determination by cryo-EM, synthetic Fabs were generated using phage display mutagenesis against ACKR3·CXCL12 complexes reconstituted in nanodiscs (NDs). Two Fabs that bound to opposite sides of the complex were chosen to aid the structure determination process. CID25 binds to an extracellular epitope that includes elements from both ACKR3 and CXCL12, and CID24 binds to the cytoplasmic pocket of the active receptor (Fig. 1A). Fab binding was confirmed by size exclusion chromatography (SEC) and an increased melting temperature (*T*_m_) of ACKR3 when in complex with the Fab (fig. S1). A nanobody that restricts the flexibility of the elbow arm between the variable and constant domains of the light chain (*15*) was used in three of the structures, although modeled in only two due to low-quality density in one case (fig. S2 and tables S1 and S2).

### Structure of the CID25–ACKR3·CXCL12 _WT_–CID24 complex

The 3.8-Å model of the CID25–ACKR3·CXCL12_WT_–CID24 complex (Fig. 1A and fig. S3, A and B) includes residues 26 to 329 of the receptor, CXCL12_WT_, and the variable regions of the two Fabs. The globular domain of the chemokine interacts with the receptor N terminus and extracellular loops (ECLs), whereas its N terminus penetrates deep into the receptor orthosteric pocket (Fig. 1, B and C). The complementarity-determining region 3 (CDR-H3) loop of the extracellular CID25 Fab forms extensive interactions with ECL2 of ACKR3, burying 2100-Å^2^ accessible surface area of CXCL12_WT_ (Fig. 1C), consistent with its ability to slow the chemokine off-rate by ~5-fold (fig. S4). In the CID25–ACKR3·CXCL12_WT_–CID24 complex, as well as the other six ACKR3 structures, no density was observed for the anticipated disulfide bond between Cys^34^ in the N terminus and Cys^287^ in ECL3 of ACKR3, despite being observed in all other reported chemokine receptors. The apparent absence of a disulfide bond at this position is consistent with the observation that mutation of these cysteines has little effect on ACKR3 function, in contrast to most canonical chemokine receptors (*16*). Density likely corresponding to cholesteryl hemisuccinate (CHS) was observed in shallow pockets on the membrane-spanning domain of the receptor (Fig. 1A, fig. S5A, and movie S1) and was modeled as cholesterol. The most prevalent cholesterol-binding site was formed by transmembrane helices (TM) 2, 3, and 4 and centered on the highly conserved W169^4.50^ side chain (fig. S5B; superscript corresponds to the Ballesteros-Weinstein numbering scheme for GPCRs). A cholesterol molecule in the analogous site is also present in structures of CXCR2 and other class A GPCRs (*17*, *18*). The second most common site is close to intracellular loop 2 (ICL2) and was observed in five structures (fig. S5C), whereas a site close to the orthosteric pocket is evident in three structures (fig. S5D). Depletion of membrane cholesterol reduces chemokine binding and signaling of CXCR4 and CCR5 (*19*, *20*), although whether any of these sites regulate the activity of ACKR3 or other chemokine receptors remains to be determined.

**Fig. 2.**
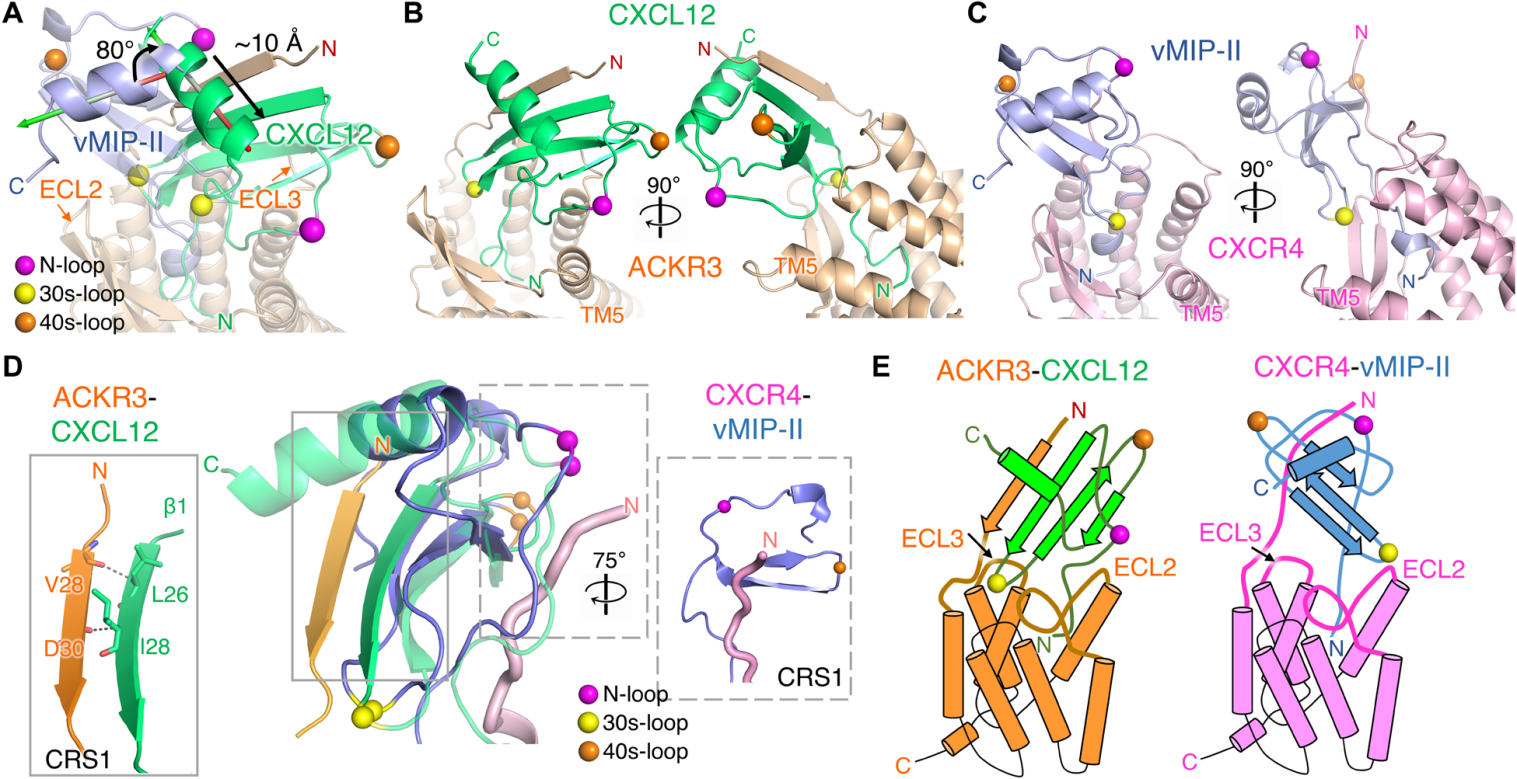
Atypical binding mode of CXCL12 on ACKR3. (**A**) Superposition of receptor subunits from the structures of CID25-ACKR3·CXCL12_WT_-CID24 (PDB entry 7SK3) and CXCR4 in complex with vMIP-II (PDB entry 4RWS). Orthogonal views of (**B**) ACKR3·CXCL12_WT_ and (**C**) CXCR4·vMIP-II. The chemokine N-loop, 30s loop, and 40s loop are demarked by spheres (purple, yellow, and orange, respectively) for reference. (**D**) Detailed interactions in CRS1 between ACKR3·CXCL12_WT_ relative to CXCR4·vMIP-II. For the central overlay, CXCL12 and vMIP-II were superposed and only the N termini of the receptors (orange cartoon for ACKR3 and violet tube for CXCR4) are shown. (**E**) Cartoon models of the distinct receptor-chemokine complexes.

Notably, CXCL12 binds to ACKR3 in a distinctive way relative to all other reported receptor-chemokine complexes (Fig. 2). For example, compared to vMIP-II bound to CXCR4, CXCL12_WT_ is rotated by 80° and shifted by ~10 Å toward TM5 and TM6, allowing it to interact primarily with the N terminus of TM5 and ECL3, rather than with the extended ECL2 (Fig. 2A and fig. S6, A to C). Furthermore, ECL1 of ACKR3 shifts in toward the central axis of the receptor by up to 5 Å, while ECL2 moves outward by up to 5 Å (fig. S6D). The unexpected orientation of CXCL12 enables the β1 strand of the chemokine (26-LKILN-30) to form a parallel β-strand interaction with N-terminal residues 28-VVDTVMC-34 of ACKR3. This contrasts with canonical chemokine receptors wherein the receptor N terminus binds in a shallow groove flanked by the chemokine “N-loop” and “40s-loop,” defined as chemokine recognition site 1 (CRS1; Fig. 2, B to E). Regardless, the CXCL12 N terminus still penetrates the ACKR3 orthosteric pocket [chemokine recognition site 2 (CRS2)], as deeply as the N termini of chemokines in structures of CCR5-CCL3 (*21*) and CXCR2-CXCL8 (fig. S7) (*17*).

**Fig. 3.**
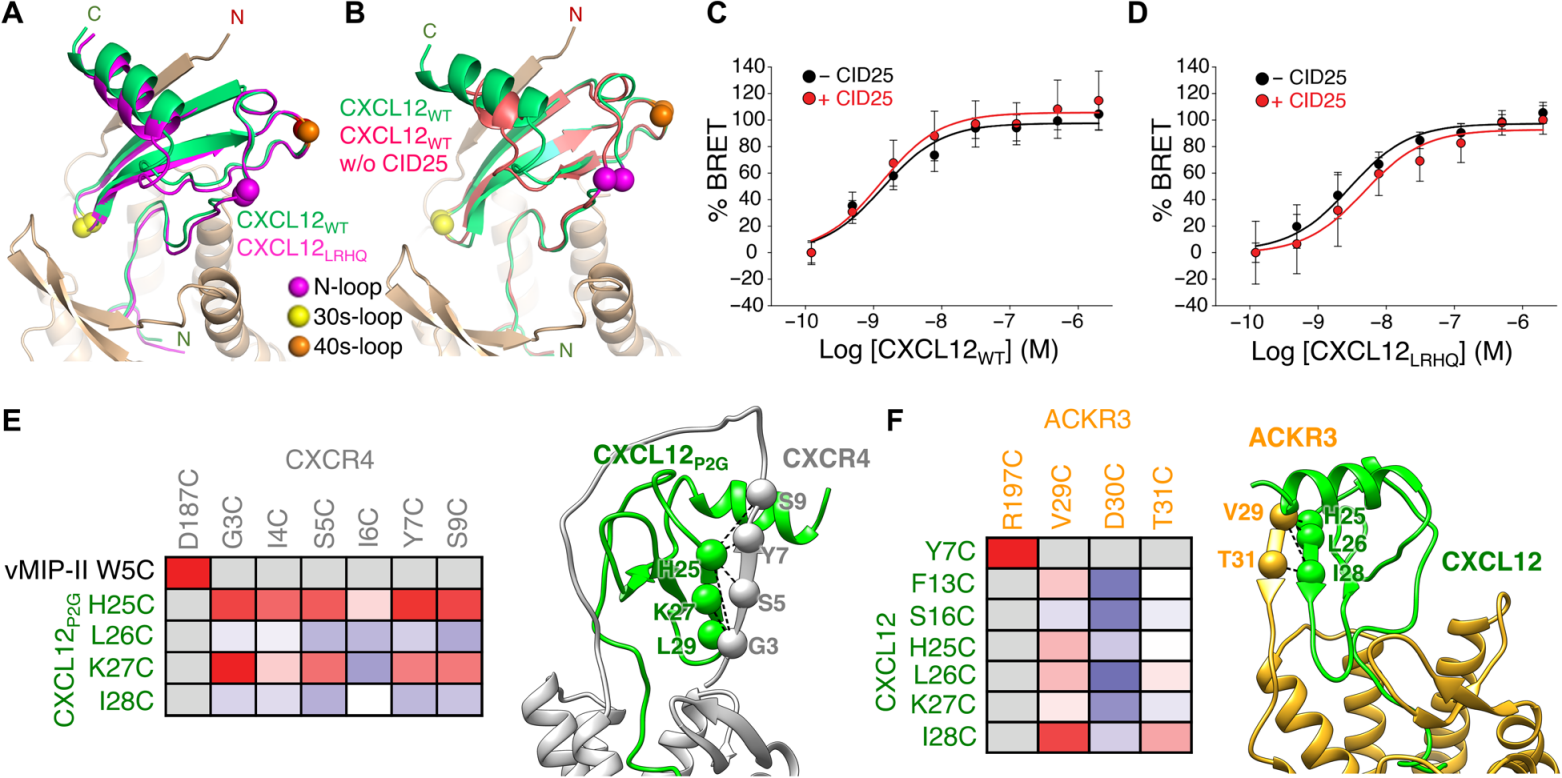
The binding mode of CXCL12 on ACKR3 is not altered by CID25 and is distinct from that of CXCR4. (**A**) Comparison of CID25-ACKR3·CXCL12_WT_-CID24 (PDB entry 7SK3) with CID25-ACKR3·CXCL12_LRHQ_-CID24 (PDB entry 7SK4) reveals a nearly identical mode of interaction. (**B**) Comparison of CID25-ACKR3·CXCL12_WT_-CID24 with ACKR3·CXCL12_WT_-CID24-NB (PDB entry 7SK5) reveals that CID25 does not greatly influence the chemokine pose. (**C** and **D**) Bioluminescence resonance energy transfer (BRET) assays of GFP10–β-arrestin 2 recruitment to ACKR3-RlucII as a function of CXCL12_WT_ (C) or CXCL12_LRHQ_ (D) in the absence or presence of 2 μM CID25. The extracellular Fab had an insignificant effect on the negative logarithm of the half maximal effective concentration (−8.87 and −8.89 without or with CID25 for CXCL12_WT_, respectively, *P* > 0.5; −8.53 and −8.32 without or with CID25 for CXCL12_LRHQ_, respectively, *P* > 0.3). The *E*_max_ was also not affected by CID25 for either chemokine (*P* > 0.05). Statistics were determined by extra sum-of-squares *F* test. Data are a composite of three separate experiments measured in triplicate and normalized to the −CID25 dataset for each ligand. Error bars correspond to SD across the three experiments. (**E** and **F**) Disulfide cross-linking of ACKR3 and CXCR4 with CXCL12 confirms that the two complexes have distinct chemokine binding poses. Heatmaps depicting the relative cross-linking efficiency from coexpression of single cysteine mutants of CXCL12 and ACKR3 versus CXCR4 and CXCL12_P2G_ [a point mutant of CXCL12 that functions as an antagonist (*41*)] in *Sf9* cells quantified by flow cytometry (table S3). The cross-link propensity is depicted from blue (absent) to red (strong). Positive disulfide cross-links are noted with spheres and dashed lines on the accompanying structures. The CXCR4 cross-linking data were adapted from (*22*).

We also determined the 3.3-Å structure of the CID25-ACKR3·CXCL12_LRHQ_-CID24 complex. CXCL12_LRHQ_ is a variant selected by phage display that has three amino acid substitutions at its N terminus along with a one-residue extension. It forms a more stable complex with ACKR3 than CXCL12 (*12*) and has a slower off-rate (*14*). CXCL12_LRHQ_ binds to ACKR3 in the same unique orientation as CXCL12_WT_ despite differences in interactions with the orthosteric pocket (Fig. 3A), suggesting structural independence of the CRS1 and CRS2 sites.

Given the extensive interactions of CID25 with both ACKR3 and CXCL12, we tested whether CID25 influenced the ligand pose by determining cryo-EM structures in its absence. The resulting 4-Å maps [Protein Data Bank (PDB) entries 7SK5 and 7SK6] indicated that CXCL12_WT_ retains the unique binding pose, although it is slightly shifted and more dynamic in the absence of CID25 (Fig. 3B and fig. S8). Recruitment of β-arrestin to ACKR3 in response to either chemokine variant is unaffected by the presence of CID25 (Fig. 3, C and D), further indicating that the extracellular Fab does not alter the function of the complex. Last, to further establish that CXCL12 binds to ACKR3 in a manner distinct from CXCR4 (because there is yet no reported structure of CXCR4·CXCL12), we used disulfide cross-linking to trap CXCL12 in its complex with ACKR3 in cells for comparison with similar disulfide cross-linking data previously collected on CXCL12_P2G_ in its complex with CXCR4 (Fig. 3, E and F). Strong cross-links observed between CXCL12-I28C and ACKR3-V29C and ACKR3-T31C are consistent with the observed orientation of CXCL12 in the ACKR3 complex. Stronger cross-links between ACKR3-V29C and ACKR3-T31C with CXCL12-L26C and CXCL12-I28C compared to CXCL12-K27C further support the register of the parallel β strand built into our cryo-EM maps. In contrast, the CXCR4·CXCL12_P2G_ disulfide cross-linking data are consistent with CXCL12_P2G_ binding to CXCR4 in a canonical orientation, with the strongest cross-links forming between CXCL12_P2G_-L29C in the β1 strand and CXCR4-G3C in the distal receptor N terminus (*22*). Also, consistent with ACKR3 forming CRS1 with the β1 strand of CXCL12, it has been shown that CXCL12 dimers, which assemble via the chemokine β1 strand, have greatly reduced affinity for ACKR3 [*K*_d_ (dissociation constant) > 1 μM], but readily bind CXCR4 (*K*_d_ = 28 nM; fig. S9) (*23*).

### Orthosteric binding pocket interactions

**Fig. 4.**
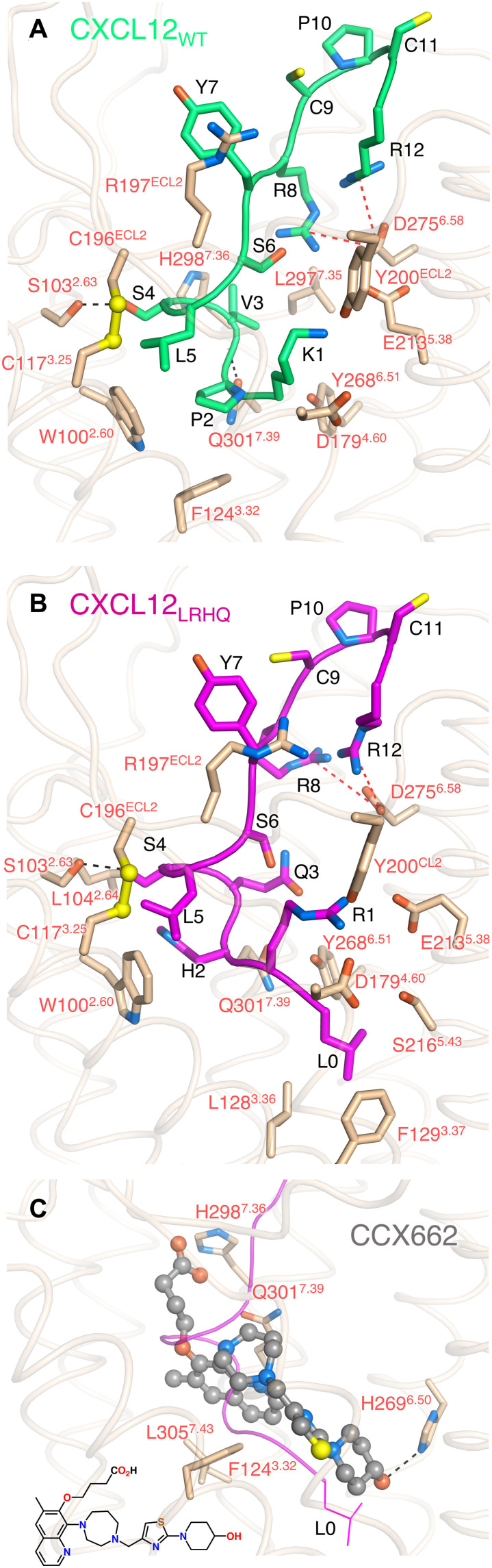
Interactions within the orthosteric pocket of ACKR3. CXCL12_WT_-K1 through CXCL12_WT_-R12 are shown with green carbons (**A**), CXCL12LRHQ-L0 through CXCL12_LRHQ_-R12 are shown with magenta carbons (**B**), and CCX662 (ball and stick model) is shown with gray carbons (**C**). In (C), a magenta backbone trace of CXCL12_LRHQ_-L0 through CXCL12_LRHQ_-R12 is shown for comparison, and the chemical structure of CCX662 is shown for reference. ACKR3 residues that contact the ligands are shown as sticks and colored based on atom type. Sulfur atoms involved in disulfide bond are shown as yellow spheres. Black and red dashed lines indicate putative hydrogen bonds and ionic interactions, respectively.

In CRS2, the N terminus of CXCL12 primarily occupies the minor orthosteric pocket formed by TM1, TM2, TM3, and TM7 of the receptor, with the exception of CXCL12-K1, which reaches into the major receptor binding pocket (formed by TM3 to TM7), placing its side chain near the side chains of ACKR3-E213^5.38^, ACKR3-D179^4.60^, and ACKR3-Y200^ECL2^ and its backbone carbonyl close enough to form a hydrogen bond with the hydroxyl of Y268^6.51^ (Fig. 4A). P2 of CXCL12 packs against the side chains of ACKR3-W100^2.60^ and ACKR3-F124^3.32^, whereas the side chain of CXCL12-V3 packs against ACKR3-L297^7.35^ and ACKR3-H298^7.36^, and its backbone amide forms a hydrogen bond with ACKR3-Q301^7.39^. CXCL12-S4 potentially forms a hydrogen bond with the hydroxyl group of ACKR3-S103^2.63^. The side chain of CXCL12-L5 sits in a hydrophobic patch below the conserved C117^3.25^-C196^ECL2^ disulfide bond, Y7 is in proximity to R197^ECL2^, and the side chains of R8 and R12 engage D275^6.58^. These interactions are consistent with alanine mutations of ACKR3-W100^2.60^, ACKR3-F124^3.32^, ACKR3-D179^4.60^, ACKR3-R197^ECL2^, ACKR3-E213^5.38^, ACKR3-Y268^6.51^, ACKR3-D275^6.58^, and ACKR3-Q301^7.39^, which all decrease the ligand-dependent potency and/or efficacy of arrestin recruitment to ACKR3 [(*24*, *25*) and fig. S10, A and C], as well as the fact that coexpression of Cys mutants ACKR3-R197 ^ECL2^C and CXCL12-Y7C in *Sf9* cells leads to the formation of a highly stable, disulfide-trapped, receptor-chemokine complex (*24*). CXCL12 N-terminal mutants K1R and P2G have reduced affinity for ACKR3, and truncation of the three first residues results in a complete loss of ACKR3 binding and arrestin recruitment, confirming the functional importance of these residues (*14*, *26*).

Despite having an extra residue (L0) at the N terminus followed by three residue substitutions (RHQ) relative to CXCL12_WT_, CXCL12_LRHQ_ recruits arrestin with unchanged potency and efficacy (*14*). In the orthosteric pocket, the backbone of CXCL12_LRHQ_ aligns with CXCL12_WT_ and makes many of the same receptor-chemokine interactions (Fig. 4B). The major difference is that CXCL12_LRHQ_-L0 packs in a hydrophobic pocket formed by the side chains of ACKR3-L128^3.36^, ACKR3-F129^3.37^, and ACKR3-S216^5.43^, which likely explains the slower dissociation of CXCL12_LRHQ_ relative to CXCL12_WT_. The CXCL12_LRHQ_-R1 side chain is oriented similarly to that of CXCL12_WT_-K1, whereas the CXCL12_LRHQ_-H2 side chain extends into a pocket formed by ACKR3-W100^2.60^, ACKR3-L104^2.64^, and ACKR3-Q301^7.39^, which may also contribute to the slower off-rate.

A small-molecule agonist, CCX662, was included in our early solubilizations of ACKR3 and was initially thought to wash out before assembling complexes with CXCL12_WT_. However, we observed density for the agonist in the cryo-EM maps, resulting in the first six residues of CXCL12_WT_ being disordered in both the NB-CID25-ACKR3·CXCL12_WT_·CCX662 and CID25-ACKR3·CXCL12_WT_·CCX662-CID24 complexes. To eliminate any ambiguity, we also determined the 3.8-Å structure of ACKR3·CCX662-CID24. In each of the three structures, CCX662 binds in the orthosteric pocket, with its core occupying a similar volume as the first few residues of CXCL12_LRHQ_ (Fig. 4C). The 4-hydroxypiperidine ring of CCX662 projects deepest into the pocket, where its hydroxyl group is positioned to form a hydrogen bond with the side chain of H269^6.52^. Both CXCL12_LRHQ_ and CCX662 appear to induce a similar 1- to 2-Å outward shift of TM5 residues 212 to 219 relative to CXCL12_WT_, likely due to the packing interactions formed by L0 and the hydroxypiperidine ring, respectively. The thiazole and homopiperazine rings of CCX662 effectively mimic the backbone interactions of L0 through H2 of the chemokine. However, its bicyclic aromatic ring occupies a distinct pocket bounded by the side chains of ACKR3-F124^3.32^, ACKR3-Q301^7.39^, and ACKR3-L305^7.43^. The poorly ordered carboxylic acid moiety of CCX662 is positioned so that it could interact with either ACKR3-S103^2.63^, ACKR3-N108^ECL1^, or ACKR3-H298^7.36^ (fig. S3C). When compared to the chemokine-bound structures, several ACKR3 side chains are rearranged in the CCX662 complex. For example, ACKR3-Q301^7.39^ is reoriented toward the entrance of the pocket and forms no hydrogen bonds with CCX662, and the H121^3.29^ side chain adopts two distinct conformers. Still, CCX662 recruits arrestin with almost equal efficacy as CXCL12 (*14*), consistent with the ready activation of ACKR3 once its binding pocket is filled. CCX662 maintains selectivity for ACKR3 over CXCR4 because the analogous contacting residues in the CXCR4 orthosteric pocket tend to be bulkier and would sterically occlude binding. For example, ACKR3-S216^5.43^ and ACKR3-L305^7.43^ are substituted by CXCR4-H203^5.43^ and CXCR4-F292^7.43^, respectively.

### ACKR3 exhibits many of the key signatures of an active GPCR, consistent with constitutive activity

**Fig. 5.**
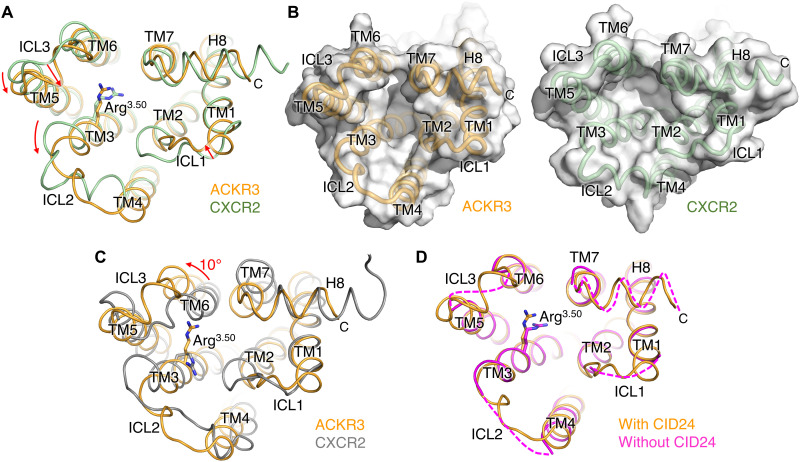
The TM helices of ACKR3 adopt an overall configuration most similar to that of an active GPCR. (**A**) Intracellular view of ACKR3 in the CID25-ACKR3·CXCL12_WT_-CID24 complex (PDB entry 7SK3) superimposed with the active state structure of CXCR2 in complex with CXCL8 and Gα_i_Gβ_1_γ_2_ (PDB entry 6LFO). Arrows indicate major differences in TM helices or intracellular loops in ACKR3 relative to those of CXCR2. (**B**) Side-by-side surface representations of the cytoplasmic cleft of ACKR3 and CXCR2 from (A) to highlight the smaller cleft of ACKR3 relative to CXCR2, which is due chiefly to inward movement of ICL1 and a more subtle change in H8. Note also the lack of a kink at the N terminus of the TM4 helix in ICL2, which along with the smaller cleft, could be a determinant of bias. Thus the conformation of TM4 more closely resembles those of inactive chemokine receptors [see (C)]. (**C**) Intracellular view of ACKR3 in the CID25-ACKR3·CXCL12_WT_-CID24 complex (PDB entry 7SK3) superimposed with inactive CXCR2 in complex with antagonist 00767013 (PDB entry 6LFL). TM6 in ACKR3 is rotated outward by 10° compared to inactive CXCR2, whereas TM3 and TM4, which bracket ICL2, are similarly extended, unlike in 6LFO (A). (**D**) Intracellular view of ACKR3 determined in the presence (PDB entry 7SK3) or absence (PDB entry 7SK7) of intracellular Fab CID24. Dashed lines indicate connections that could not be reliably modeled. The side chains of Arg^3.50^ are shown in each panel to demonstrate its distinct conformation in the compared structures.

Despite the apparent inability of ACKR3 to activate G proteins (*3*, *27*), the position of its TM helices in all our structures resembles those of active-state chemokine receptors, such as CXCR2 in complex with CXCL8 and Gα_i_Gβ_1_γ_2_ [0.9 Å root mean square deviation (RMSD) for 174 Cα atoms; Fig. 5, A and B] (*17*). In contrast, the RMSD upon comparison to an inactive CXCR2 structure is much larger (1.9 Å for Cα 186 atoms; Fig. 5C). The open, active state–like configuration of the intracellular cleft accommodates CDR-H3 of CID24, burying 2100 Å^2^ of accessible surface area (Fig. 1D). CID24 binding further stabilizes the complex by slowing the off-rate of the chemokine (fig. S4), similar to the allosteric effect of G proteins on the agonist off-rate of the β_2_AR (*28*). However, the intracellular loops of ACKR3 exhibit large differences in both sequence and structure from canonical chemokine receptors, including the presence of a short helix in ICL3 and the lack of a kink at the N terminus of TM4 in ICL2 (Fig. 5, A and B). To test the possibility that CID24 perturbed the conformation of these loops, we determined the 3.3-Å structure of the ACKR3·CXCL12_WT_·CCX662 complex in the absence of CID24 (table S1), which may be the first structure of a receptor in an active-state conformation without cytoplasmic cleft binding partners or stabilizing crystal contacts. The open active-like conformation of the TM helices is maintained, but all the ICLs and H8 exhibit disorder (Fig. 5D). The conformational heterogeneity of these loops is consistent with the dynamic nature expected for class A family GPCRs in their active state (*29*). Furthermore, maintenance of an open cleft conformation in the absence of CID24 suggests that extracellular ligand binding is sufficient to stabilize an active-like configuration of the ACKR3 TM helices.

In addition to the general configuration of the TM helices, all the “microswitches” (*30*) in the ACKR3 structures have hallmarks typical of active canonical chemokine receptors. This includes the NPxxY motif in TM7 and the orientation of residue 5.58, which typically interacts with residue 3.50 of the conserved “DRY” motif. In the absence of CID24, the density is consistent with R142^3.50^ forming a hydrogen bond with Y232^5.58^, reproducing a stabilizing interaction that forms in active class A GPCR structures (*31*, *32*). The orientations of PIF motif residues in positions 3.40, 5.50, and 6.44, and W265^6.48^ of the conserved CWxP motif located below the orthosteric pocket, are also consistent with canonical active receptor conformations. Unlike CXCR4, ACKR3 constitutively internalizes and recycles to the plasma membrane as it scavenges CXCL12 (*7*) and it also constitutively associates with β-arrestin2 (*33*). The active-like configuration of the TM helices and microswitches of ACKR3 likely contributes to its constitutive association with β-arrestin2, but whether it also contributes to constitutive trafficking remains to be determined.

**Fig. 6.**
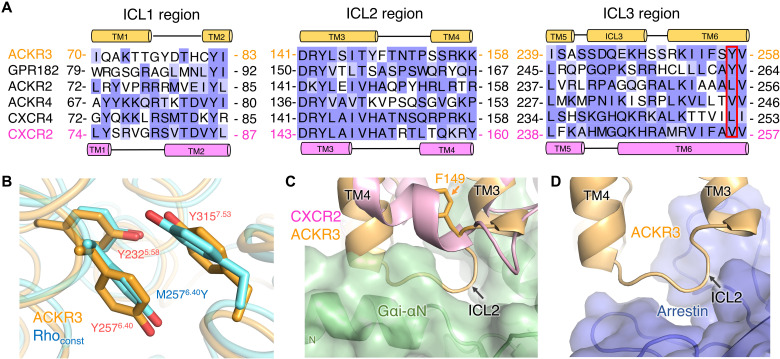
Residues within the intracellular cleft of ACKR3 assume an active-like conformation, but ICL2 assumes an inactive configuration that seems incompatible with G protein coupling. (**A**) Sequence alignment of ICLs in atypical chemokine receptors versus CXCR4 and CXCR2. Secondary structures are shown on top and bottom for ACKR3 and CXCR2, respectively. Positions corresponding to ACKR3-Y257^6.40^ are bounded with a red box. ACKR1 is omitted because of its high sequence divergence. (**B**) Orientation of ACKR3-Y232^5.58^, ACKR3-Y257^6.40^, and ACKR3-Y315^7.53^ (PDB entry 7SK3, orange) compared with the N2C/M257^6.40^Y/D282C constitutively active rhodopsin mutant (Rho_const_; PDB entry 4A4M, cyan). (**C**) Superposition of ACKR3 (PDB entry 7SK3, orange) with CXCR2 in complex with CXCL8 and Gα_i_Gβ_1_γ_2_ (PDB:6LFO, CXCR2 in pink, Gα_i_ in green). The nonconserved side chain of F149 in ICL2 of ACKR3 [orange sticks, see (A)] may prevent the kink that forms in TM4 of CXCR2. (**D**) Superposition of ACKR3 (PDB entry 7SK3, orange) with the M2 muscarinic receptor–arrestin complex (PDB:6U1N; only arrestin is shown in blue).

To understand why ACKR3 adopts an active-like conformation, we aligned the sequence of ACKR3 with other chemokine receptors, which highlights a Tyr residue (Y257^6.40^) on the intracellular side of TM6 at a position where all other chemokine receptors have shorter hydrophobic side chains (Fig. 6A) (*34*). Mutation of the 6.40 position of CXCR1 (V247^6.40^N) and rhodopsin (M257^6.40^Y) increases constitutive receptor activity and, in the case of CXCR1, affinity for bound chemokine (*35*); the latter effect is suggestive of positive allostery similar to G protein stabilization of agonist binding (*28*). In the ACKR3 structure, the side chain of Y257^6.40^ packs with Y232^5.58^ and Y315^7.53^ of the NPxxY microswitch forming a π-π interaction. The orientation of these three Tyr side chains in ACKR3 is analogous to the corresponding residues in the constitutively active N2C^NT^/M257^6.40^Y/D282C^ECL3^ rhodopsin mutant (Fig. 6B) (*36*). Thus, we hypothesize that Y257^6.40^ contributes to constitutive receptor activity by stabilizing the interactions of TM5, TM6, and TM7 in their active configuration. Consistent with this hypothesis, mutation of Y257^6.40^ to Leu, the corresponding residue in CXCR4, led to reduced constitutive and ligand-induced activity (fig. S10, B and C). Similarly, the Y315^7.53^A mutation reduced chemokine affinity and impaired the ability of ACKR3 to recruit arrestin (*37*).

### Features that could play a role in intrinsic bias against G proteins

Because CCX662 can efficiently recruit arrestin but does not activate G proteins (fig. S10D), arrestin bias probably has little to do with the unique pose observed for CXCL12 in the ACKR3 complexes (*14*). Moreover, the configuration of the TM helices and the microswitch signatures of ACKR3 are consistent with active-state structures of canonical GPCRs. However, the ICLs of CID24-bound ACKR3 are markedly different from those of active-state CXCR2 (*17*). A kink at the beginning of CXCR2 TM4 in the ICL2 region seems necessary to accommodate the N-terminal helix of Gα. ACKR3 structures with and without CID24 lack this kink, and ICL2 would likely collide with Gα based on the superposition of these structures with the structure of active-state CXCR2 (Fig. 6C). On the other hand, comparison with arrestin-bound GPCRs suggests that the ICL2 loop of ACKR3 would still accommodate arrestin in a “receptor-core” binding mode. The observed helix in ACKR3 ICL3 might also discourage arrestin binding, but this loop is disordered in the absence of CID24 and thus likely can adapt to whatever protein is bound (Fig. 6D). We therefore attempted to test whether the ICLs were responsible for arrestin bias by creating chimeras of ACKR3, wherein one or all of its ICLs were exchanged with those of CXCR2. However, all but the ICL1 swap were severely impaired in arrestin recruitment, suggesting defects in expression, folding, or transport, and none of these chimeras activated G protein (fig. S11). Notably, the conformations of ICL1 and ICL2 as predicted by AlphaFold2 (*38*) were similar to those observed in the CID24-bound complexes (fig. S12), suggesting that they are an intrinsic feature of the primary sequence and not a consequence of the bound Fab. Another feature that may contribute to arrestin bias is the cytoplasmic pocket, which is more compact in ACKR3 compared to active CXCR2 (Fig. 5, A and B), with inward movements of TM3, TM5, ICL1, and ICL3 despite a similar ~10° outward rotation of TM6 relative to the inactive CXCR2 structure (Fig. 5C).

## DISCUSSION

Beyond determining the basic architecture of ACKR3 and how it interacts with CXCL12, a goal of this work was to gain insight into the atypical promiscuous activation of ACKR3 by diverse ligands. To date, a nanobody and a small-molecule antagonist are the only known ACKR3 ligands that do not promote arrestin recruitment (*39*, *40*). This susceptibility to activation contrasts with CXCR4 for which most ligands except the native CXCL12 are antagonists, and even single-site mutations of CXCL12 (e.g., CXCL12_P2G_) convert the chemokine into an antagonist (*41*). For example, orthosteric pocket mutations Y116^3.32^A and D187^ECL2^A and even conservative substitutions such as D97^2.63^N and E288^7.39^Q effectively abolish arrestin recruitment to CXCR4, indicating that it requires precise receptor-chemokine interactions (e.g., CXCL12-K1 with CXCR4-E288^7.39^ and CXCR4-D97^2.63^) (*42*). In contrast, no residues that completely block arrestin recruitment to ACKR3 have been identified. Instead, mutation of ACKR3-Q301^7.39^ (corresponding to CXCR4^−^E288^7.39^), and to a lesser extent ACRK3-Y268^6.51^, results in increased constitutive activity (fig. S10A). Thus, it seems that distortion of the binding pocket by steric bulk may be sufficient for ACKR3 activation, as has been suggested for the viral chemokine receptor US28 (*43*). Plasticity of the receptor, as exhibited by the conformational heterogeneity of some of the side chains (e.g., Q301^7.39^ and H121^3.29^) as well as a segment of TM5 at the base of the orthosteric pocket, may facilitate binding of diverse ligands and allow for distortion-driven activation. The absence of a disulfide bond between the receptor N terminus and ECL3 also likely contributes to the deformability of the orthosteric pocket by diverse ligands.

Another major goal of this study was to identify possible molecular mechanisms underlying receptor bias. A confounding factor in this analysis was that the complexes were stabilized by Fab fragments that could influence the structure of bound chemokine or of the ICLs that engage G proteins. We addressed these concerns by determining ACKR3 structures in the absence of either the extracellular or intracellular Fab. In these structures, the underlying conformations of ACKR3 and CXCL12 remained fundamentally the same, although the regions that bound to the Fabs became more dynamic. We therefore conclude that the intrinsic properties of the ICL regions probably play a key role in dictating bias, in particular the lack of a kink at the cytoplasmic end of TM4 in ICL2. The structures of ACKR3 may thus be analogous to a GPCR in a “partially active state” (*44*). It makes sense that the configuration of the ICL2 region would be a driver of bias because this element directly interacts with heterotrimeric G proteins in many of the active class A receptor structures determined to date (*45*) and modeling does not suggest that its conformation would interfere with GRK (*46*) or arrestin binding to ACKR3. Comparison of the sequence of ICL2 with other ACKRs, which also do not couple to G proteins, does not reveal a high degree of conservation in ICL2 or residues analogous to ACKR3-F149 (Fig. 6A). The only other ACKR predicted by AlphaFold2 to adopt a similar “partially active” ACKR3-like TM configuration is GPR182. Its modeled ICL2 loop, although distinct from that of ACKR3, would also collide with heterotrimeric G proteins, and GPR182 similarly retains a Y^6.40^ side chain. It is possible that other ACKRs could still impose bias in the same manner as ACKR3 but with unique ICL2 structures. In support of ICL2 playing a role in selecting for arrestin, double electron–electron resonance spectroscopy has shown that ICL2 is involved in large structural transitions imposed by arrestin-biased agonists of the angiotensin II type 1 receptor (*47*). Another factor contributing to arrestin bias in ACKR3 could be the smaller size of its cytoplasmic cleft (Fig. 5, A and B), as others have suggested (*44*, *47*). However, GPCRs can probably achieve a more compact arrestin-selective cytoplasmic cleft in various ways, including via conformational changes at the cytoplasmic end of TM7 (*48*).

Although we and others have failed to detect G protein activation in human embryonic kidney (HEK) 293 (fig. S10D) and other cells (*4*, *27*), data from a bioluminescence resonance energy transfer (BRET) based G protein recruitment assay suggest that ACKR3 is at least in close proximity with G proteins in cells (fig. S13), consistent with other reports (*4*, *33*). A lack of coupling may occur if ACKR3 and G proteins colocalize on membranes, but do not directly interact. Alternatively, if G proteins can still engage with ACKR3 via ICL3, Arg142^3.50^, and H8, but fail to productively engage ICL2, then we predict that nucleotide exchange and G protein activation would be greatly diminished (*45*). G protein recruitment is not enhanced by the addition of CXCL12 (fig. S13). This directly contrasts with the enhancing effect of CXCL12 on CXCR4–G protein association (*4*) and suggests that the ACKR3 interaction with G proteins, if any, is noncanonical. If the ability to bind but not activate G proteins is ultimately confirmed, it could represent yet another mechanism by which ACKR3 negatively regulates CXCR4 (*4*). On the other hand, there are reports that ACKR3 can activate Gα_i/o_ proteins in astrocytes and glioma cells (*33*, *49*), hinting that the specific cellular environment could play a role in dictating the bias of ACKR3. For example, interactions with specific proteins or lipids, unique membrane environments, or posttranslational modifications could influence the preferred conformations of ACKR3. The observed structural plasticity of ACKR3 may facilitate these possibilities.

In the future, it would be instructive to examine structures of ACKR3 with other chemokines such as CXCL11 to determine whether the binding pose of CXCL12 on ACKR3 is unique. Structures of the receptor bound to heterotrimeric G proteins, if they indeed form stable and specific complexes, would be of interest to examine the mechanism by which activation of G proteins can be uncoupled from binding. Specific complexes of ACKR3 with GRKs and arrestins would also provide important insights into further understanding the function and signaling mechanisms of ACKR3.

## MATERIALS AND METHODS

### Expression and purification of ACKR3-chemokine complexes in *Sf9* cells

Human ACKR3 was expressed in *Sf9* cells as previously described (*50*). Briefly, ACKR3 (residues 2 to 362) and ACKR3Δ332 (residues 2 to 332) with an N-terminal hemagglutinin (HA) signal sequence and C-terminal FLAG and His tags were cloned into a pFastBac1 vector under a GP64 promoter. Human CXCL12 was cloned after a polH promoter in the pFastBac1 vector. Mutagenesis was performed using standard site-directed mutagenesis techniques (QuikChange and overlap extension) and confirmed by Sanger sequencing.

Production of baculoviruses (ACKR3, CXCL12, and mutants thereof) was performed using the Bac-to-Bac Baculovirus Expression System (Invitrogen) as described previously (*50*). pFastBac1 vectors containing ACKR3 or CXCL12 were transformed into DH10Bac cells and plated onto LB agar plates containing kanamycin (50 μg/ml), gentamicin (7 μg/ml), tetracycline (10 μg/ml), Bluo-Gal (100 μg/ml), and isopropyl-β-d-1-thiogalactopyranoside (IPTG; 40 μg/ml; Teknova). After 48 hours, single white colonies were picked and grown overnight in 5 ml of LB medium with kanamycin (50 μg/ml), gentamicin (7 μg/ml), and tetracycline (10 μg/ml). *Sf9* cells were cultured in ESF 921 medium (Expression Systems) at 27°C with shaking at 140 rpm. Bacmid DNA was isolated and mixed with transfection medium and X-tremeGene HP DNA for 30 min at room temperature before addition to 2.5 ml of 2.0 × 10^6^/ml *Sf9* cells in a 24–deep well plate. The transfection mixture was then incubated at 27°C for 96 hours while shaking at 300 rpm. After 96 hours, the plates were centrifuged (20 min at 2000*g*) and the P0 virus containing supernatant was collected. Four hundred microliters of P0 virus was added to 40 ml of 2.0 × 10^6^/ml *Sf9* cells and incubated at 27°C with shaking at 130 rpm for 48 hours, after which the cells were again spun down (20 min at 2000*g*), and the P1 virus–containing supernatant was collected and stored at 4°C. Expression of ACKR3 or coexpression of ACKR3 and chemokine was achieved by adding P1 virus to *Sf9* cells at a density of 2 × 10^6^ to 3 × 10^6^/ml with a multiplicity of infection (MOI) of 6 for each virus. After 48 hours of infection, cell pellets were collected and stored at −80°C.

Cell pellets were thawed and dounce-homogenized 40 times in hypotonic buffer [10 mM Hepes (pH 7.5), 10 mM MgCl_2_, and 20 mM KCl]. After centrifugation for 30 min at 50,000*g*, the membrane pellets were resuspended in hypotonic buffer containing 1 M NaCl, dounced another 40 times, and repelleted. This process was then repeated two more times. After the final spin, the membranes were dounced again in hypotonic buffer with 30% glycerol and stored at −80°C until further use.

To purify ACKR3 or ACKR3·CXCL12 complexes, thawed membranes were incubated with iodoacetamide (2 mg/ml) and protease inhibitors for 30 min at 4°C, before diluting 2× with solubilization buffer [100 mM Hepes (pH 7.5), 0.8 M NaCl, 1.5%/0.3% *n*-dodecyl-β-d-maltoside (DDM)/CHS]. CCX662 (ChemoCentryx) (*13*) at 100 μM final concentration was included in samples of ACKR3 alone to stabilize the receptor (*14*). After 4 hours of solubilization, the samples were centrifuged for 30 min at 50,000*g* to remove insoluble components. Talon resin (Teknova) and 20 mM imidazole were then added to the soluble supernatant, and the sample was incubated overnight at 4°C. The next morning, the receptor-bound resin was pelleted by centrifugation for 5 min at 350*g* and washed with 25 ml of wash buffer 1 [WB1; 50 mM Hepes, 400 mM NaCl, 0.1/0.02% lauryl maltose neopentyl glycol (LMNG)/CHS, 10% glycerol, 20 mM imidazole (pH 7.5)] and centrifuged again. The resin was then transferred to Poly-Prep Chromatography columns (Bio-Rad) and washed with an additional 20 ml of WB1, followed by 20 ml of WB2 [50 mM Hepes, 400 mM NaCl, 0.025/0.005% LMNG/CHS, 10% glycerol, and 20 mM imidazole (pH 7.5)], and finally eluted with elution buffer (WB2 with 250 mM imidazole). The elutions were concentrated, and imidazole was removed with a desalting column and then buffer exchanged to low-salt buffer [50 mM Hepes (pH 7.5), 100 mM NaCl, 0.025/0.005% LMNG/CHS, 10% glycerol]. Protein concentrations were determined by *A*_280_ using extinction coefficients of 77,000 and 85,000 M^−1^ cm^−1^ for ACKR3 and ACKR3·CXCL12 complexes, respectively. Receptors were aliquoted, snap-frozen, and stored at −80°C until use.

### Expression and purification of chemokines in *Escherichia coli*

CXCL12_WT_ and CXCL12_LRHQ_ were expressed and purified as previously described (*24*). Briefly, chemokines in pMS211 plasmids were transformed into BL21(DE3)pLysS *Escherichia coli* cells and grown to OD_600_ (optical density at 600 nm) of 0.6 in LB medium. Expression was induced with 1 mM IPTG, and cells were grown for another 6 hours before harvesting by centrifugation for 10 min at 6000*g*. The resulting cell pellet was resuspended in lysis buffer [50 mM tris (pH 7.5) and 150 mM NaCl] and sonicated at room temperature, and soluble materials were removed by centrifugation at 12,000*g* for 15 min, after which the pellet was again dissolved in lysis buffer and inclusion bodies were collected by centrifugation at 6000*g* for 15 min. The inclusion bodies were resuspended by dounce homogenization in equilibration buffer [50 mM tris, 6 M guanidine-HCl (pH 8.0)] and sonicated once more to extract chemokine. Insoluble material was removed by centrifugation at 6000*g* for 15 min, and the supernatant was passed over a Ni–nitrilotriacetic acid (NTA) column equilibrated in equilibration buffer. The column was washed with equilibration buffer followed by wash buffer [50 mM MES (pH 6.0), 6 M guanidine-HCl], and the chemokine was eluted with 50 mM acetate (pH 4.0) and 6 M guanidine-HCl. The chemokine elutions were pooled, dithiothreitol was added to 4 mM final concentration, and then the sample was incubated for 10 min at room temperature before adding the solution dropwise into refolding buffer [50 mM tris (pH 7.5), 500 mM arginine-HCl, 1 mM EDTA, 1 mM oxidized glutathione]. The refolding solution was incubated with minimal stirring for 4 hours at room temperature and then dialyzed against 20 mM tris (pH 8.0) and 50 mM NaCl with two buffer changes. After dialysis, precipitate was removed by centrifugation at 8000*g* for 30 min, CaCl_2_ was added to a final concentration of 2 mM, and the sample was incubated with enterokinase at 37°C for 2 to 4 days to cleave the N-terminal purification tag. To remove uncleaved chemokine and cleaved tag, the sample was applied to a Ni-NTA column equilibrated with 50 mM tris (pH 8.0). The column was washed with 50 mM tris (pH 8.0), and cleaved chemokine was eluted with wash buffer. As a final purification step, the sample was purified on a reversed-phase C18 column equilibrated with 75% buffer A [0.1% trifluoroacetic acid (TFA)] and 25% buffer B (0.1% TFA, 90% acetonitrile) and eluted by a linear gradient of buffer B (33 to 45%). The resulting protein was lyophilized for 48 hours and stored at −80°C until use. The purity of the *E. coli* expressed chemokine was assessed by SDS–polyacrylamide gel electrophoresis followed by Coomassie staining. As shown in fig. S14, both CXCL12_WT_ and CXCL12_LRHQ_ used for biochemical assays are of high purity. The higher molecular weight band in CXCL12_LRHQ_ corresponds to uncleaved material that was not fully separated by high-performance liquid chromatography. Because this material was only used for the titration in Fig. 3D, the same amount of active cleaved CXCL12_LRHQ_ was added to both −/+CID25 samples and the conclusion that the Fab has no effect on ACKR3 activation remains valid.

### Generation of synthetic Fabs for ACKR3

Fab selections were performed with CXCL12 in complex with full-length ACKR3 and a C-terminally truncated version. ACKR3·CXCL12 and ACKR3Δ332-CXCL12 complexes were expressed and purified as described above except that DDM/CHS was substituted for LMNG/CHS in the wash and elution buffers. The final samples [in 25 mM Hepes (pH 7.5), 10% glycerol, 400 mM NaCl, and 0.025/0.005% (w/v) DDM/CHS] were concentrated to 32 and 18 μM for the ACKR3 and ACKR3Δ332 complexes, respectively. The concentration was determined from absorbance at 280 nm using the calculated extinction coefficients of the ACKR3·CXCL12-containing NDs (ε_MSP_(26,600 M^−1^ cm^−1^) × 2 + ε_ACKR3_(76,000 M^−1^ cm^−1^)/ε_ACKR3Δ332_(73,000 M^−1^ cm^−1^) + ε_CXCL12_(8730 M^−1^ cm^−1^) = ε_ND,ACKR3_(134,930 M^−1^ cm^−1^)/ε_ND,ACKR3Δ332_(137,930 M^−1^ cm^−1^).

The ACKR3Δ332·CXCL12 complex was reconstituted into biotinylated MSP1E3D1 NDs as previously described (*50*). Briefly, 1-palmitoyl-2-oleoyl-*sn*-glycero-3-phosphocholine (POPC; Avanti) and 1-palmitoyl-2-oleoyl-*sn*-glycero-3-phospho-(1′-rac-glycerol) (POPG; Avanti) were combined in chloroform in a 3:2 POPC:POPG molar ratio, dried to a thin lipid film, and solubilized with ND buffer [25 mM Hepes (pH 7.5), 150 mM NaCl] containing 200 mM cholate. MSP1E3D1 was expressed and purified using established methods as previously described (*51*). Purified MSP was dialyzed in ND buffer and incubated for 1 hour at room temperature with a fourfold molar excess of EZ-Link NHS-PEG4-Biotin (Thermo Fisher Scientific), which reacts with free amines to biotinylate MSP. The reaction was quenched through addition of 5 mM tris (pH 7.5). ACKR3Δ332·CXCL12, biotinylated MSP1E3D1, and solubilized POPC/POPG were combined to final concentrations of 10 μM, 100 μM, and 13 mM, respectively. After 30-min incubation on ice, 200 mg of Biobeads was added followed by incubation at 4°C for 16 hours. Biobeads were removed, and the samples were loaded on a Superdex 200 10/300 GL column equilibrated with ND buffer. Fractions containing purified NDs were combined, 200 μl of Talon resin was added, and the samples were incubated at 4°C. After 20 hours, the resin was transferred to the Micro Bio-Spin Column (Bio-Rad) and washed with 5 ml of ND buffer. Receptor-containing NDs were eluted with ND buffer containing 250 mM imidazole and buffer-exchanged using Amicon Ultra-0.5 100-kDa molecular weight cutoff spin concentrators. Samples were concentrated to 1.8 μM, and purified CXCL12 was added to a final volume of 10 μM to ensure saturation of the complex. Samples were aliquoted and flash-frozen.

The ACKR3·CXCL12 complex was reconstituted into egg PC:POPG (8:2 molar ratio) MSP1D1 biotinylated NDs (*51*). Excess empty NDs were removed via Ni-NTA purification. The biotinylation efficiency of NDs was assayed by pull-down on streptavidin-coated magnetic particles. Fabs were generated from a phage display library according to previously described procedures (*52*). In brief, NDs containing ACKR3·CXCL12 were used in three independent library sorting experiments using Library E [gift from S. Koide, New York University (*53*)], ACKR3·CXCL12-1D1 NDs, ACKR3·CXCL12-1D1 NDs supplemented with a molar excess of CXCL12, and ACKR3Δ332·CXCL12-E3D1 NDs. Single-point phage enzyme-linked immunosorbent assay (ELISA) was used to evaluate specificity and binding of synthetic Fab fragments to ACKR3·CXCL12 in NDs as described previously (*54*). In brief, phage supernatants were diluted 10-fold, assayed against 50 nM biotinylated ACKR3·CXCL12 NDs in the presence of a molar excess of CXCL12, and detected with horseradish peroxidase–conjugated anti-M13 monoclonal antibody (GE Healthcare, no. 27-9421-01). All assays were performed in library sorting buffer [25 mM Hepes (pH 7.5), 200 mM NaCl] supplemented with 2% (w/v) bovine serum albumin (BSA). Signals of *A*_450_ higher than 0.2 (three times higher than average background level in this assay) were considered as positive hits, and Fab fragments corresponding to those wells were sequenced. Among 144 clones tested, 21 clones were positive and 5 unique CDR sequences of Fabs were subcloned into a pRH2.2 expression vector (a gift from S. Sidhu, University of Toronto). The sequences of the Fabs used in our structure determinations are shown in fig. S15, with their CDRs highlighted in red.

### Expression, purification, and characterization of Fabs CID24 and CID25

Expression of Fabs was carried out as described previously (*55*). In brief, Fabs were grown in *E. coli* BL21 Gold cells in 2xYT medium supplemented with ampicillin (100 μg/ml) at 37°C until the OD_600_ reached 0.8 to 0.9. Expression was then induced with 1 mM IPTG for 4 hours at 37°C. Cells were harvested by centrifugation, and pellets were frozen at −80°C until further processing. For Fab purification, cells were resuspended in lysis buffer [20 mM sodium phosphate (pH 7.4), 500 mM sodium chloride, 1 mM phenylmethylsulfonyl fluoride, 2 mM deoxyribonuclease] using a high-pressure microfluidizer (Avestin) and heated to 60°C to precipitate bacterial proteins and any residual proteolytic degradation fragments of Fabs. The resulting lysate was loaded onto a 5-ml HiTrap MabSelect Sure column (GE Healthcare) equilibrated with 20 mM sodium phosphate (pH 7.4) and 500 mM NaCl, followed by washing with 10 column volumes of equilibration buffer. Fab fragments were eluted with 0.1 M acetic acid and loaded onto a 1-ml Resource S column (GE Healthcare), washed with 50 mM sodium acetate (pH 5.0), and eluted with a linear gradient (0 to 100%) of buffer containing 50 mM sodium acetate (pH 5.0) and 2 M NaCl. Fab concentration was determined from the absorbance at 280 nm using extinction coefficients of 91,260 and 89,200 M^−1^ cm^−1^ for CID24 and CID25, respectively. Fractions containing pure Fabs were pooled, dialyzed into phosphate-buffered saline (PBS), flash-frozen in liquid nitrogen, and stored at −80°C.

The effect of Fabs on the thermostability of ACKR3 in complex with CXCL12_WT_, CXCL12_LRHQ_, and the small-molecule partial agonist CCX777 (ChemoCentryx) (*13*, *24*) was tested using the cysteine-reactive 7-diethylamino-3-(40-maleimidylphenyl)-4-methylcoumarin (CPM) dye as previously described (*50*, *56*). ACKR3·CXCL12_WT_, ACKR3·CXCL12_LRHQ_, or ACKR3·CCX777 (*57*) (0.2 to 0.4 μM final concentration) and CID24, CID25 (final concentration, 1 μM), or no Fab were added to reaction buffer [25 mM Hepes (pH 7.5), 400 mM NaCl, 10% glycerol, 0.025/0.005% (w/v) DDM/CHS, 2.5 μM CPM dye], and samples were incubated for 15 min in the dark. Thermal unfolding was followed using a RotorGene Q 6-plex reverse transcription polymerase chain reaction (RT-PCR) machine (Qiagen) from the increase in CPM fluorescence upon ramping the temperature from 25° to 95°C. Melting point (*T*_m_) values were determined from the peak in the first derivative of the traces taken from melting curves analysis using the RotorGene Q software.

For characterization of ACKR3-Fab complexes via SEC, ACKR3·CXCL12_LRHQ_ was combined with Fab (CID24 or CID25) in SEC buffer [25 mM Hepes (pH 7.5), 400 mM NaCl, 10% glycerol, 0.025/0.005% (w/v) DDM/CHS] to final ACKR3 and Fab concentrations of 5.7 and 8.8 μM, respectively. After incubation for 16 hours, samples were loaded onto a Superdex 200 10/300 GL column equilibrated with SEC buffer. Elution of the complexes was followed from absorbance at 280 nm. The top four fractions of the peak corresponding to ACKR3-CXCL12_LRHQ_-Fab complex were concentrated to 100 μl.

### Cryo-EM sample preparation and data acquisition

Purified ACKR3·CXCL12_WT_, ACKR3·CXCL12_LRHQ_, or ACKR3·CXCL12_WT_·CCX662 (i.e., containing CCX662 that was not removed during purification) was mixed with Fabs at a molar ratio of 1:1.5 and incubated with anti-FLAG M2 magnetic beads (Sigma-Aldrich) at 4°C for 2 hours. The magnetic beads were then collected with a magnetic rack separator and washed with 20 column volumes of wash buffer [20 mM Hepes (pH 8), 100 mM NaCl, 0.01/0.001% (w/v) LMNG/CHS] three times. The complex was eluted with one column volume of elution buffer (wash buffer supplemented with 0.1 mg/ml 3× FLAG peptide) three times. For CXCL12-bound complexes, 60 μM CXCL12 was added to the wash and elution buffers. For samples containing the anti-Fab nanobody (*15*), the purified nanobody was added to the purified receptor complex at a 1:1.2 molar ratio and further incubated on ice for 20 min before cryo-EM sample preparation. Purified ACKR3·ligand-Fab complex (3.3 μl at ~0.2 mg/ml) was applied to glow-discharged (EasiGlow; 25 mA for 60 s) Quantifoil R1.2/1.3 300-mesh grids. After 3 s, the grids were blotted with filter paper for 3.5 s before being plunge-frozen in liquid ethane using Vitrobot MK IV (Thermo Fisher Scientific). Micrographs of the grids were collected on a Titan Krios G1 electron microscope (FEI) equipped with a post-GIF K3 direct electron detector (Gatan) and a Quantum GIF energy filter (Gatan) in the Purdue Life Sciences Cryo-EM Facility. Micrographs were collected in super-resolution mode with a pixel size of 0.54 Å, at a defocus range of 1 to 3 μm using Leginon, and 40 frames were recorded for each movie stack at a frame rate of 78 ms per frame and a total dose of 53.8 electrons/Å^2^.

### Cryo-EM data processing

The image processing flowcharts for each dataset are shown in fig. S2. Beam-induced motion was corrected and binned twofold with MotionCor2 in Appion. The motion-corrected micrographs were imported to cryoSPARC (*58*) and processed according to the standard workflow in cryoSPARC. The contrast transfer function (CTF) parameters were estimated using the CTFFIND4 module in cryoSPARC. A small set of particles were picked using blob picker to generate class averages as templates for autopicking using template picker (*58*). After a few rounds of two-dimensional (2D) classification, initial models were generated using ab initio reconstruction and the resulting 3D models were used for heterogeneous refinement in cryoSPARC (*58*). The class showing highest-resolution features was then refined using homogeneous refinement (*58*) and nonuniform refinement (*59*). Local refinement was performed using a mask generated in RELION-3 (*60*) covering the receptor and the N-terminal half of bound Fabs to further improve the resolution of complexes in PDB entries 7SK3, 7SK4, 7SK6, and 7SK8 (table S1).

### Model building and refinement

Homology models of ACKR3, CID24, and CID25 were generated using the SWISS-MODEL server (http://swissmodel.expasy.org). These models, along with crystal structures of CXCL12 (PDB entry 6SHR) and the anti-Fab nanobody (PDB entry 6WW2), were rigid body–docked into the cryo-EM map using Chimera and manually adjusted in COOT. The resulting model was refined using phenix.real_space_refine implemented in Phenix (*61*). An exception to the above process was the model building of the lower-resolution ACKR3·CXCL12_LRHQ_-CID24 complex, which was performed using molecular dynamic flexible fitting (MDFF) (*62*). The initial model of ACKR3·CXCL12_LRHQ_-CID24 was extracted from the structure of CID25-ACKR3·CXCL12_LRHQ_-CID24 and rigid body–fitted into the EM map using Chimera. The MDFF configuration files were generated using VMD (*63*). The MDFF simulation was conducted with a grid scaling value of 0.5 for 100 ps until convergence of the protein RMSD, followed by 3000 steps of energy minimization. The resulting model from MDFF was inspected and adjusted manually in COOT to generate a template for a second run of MDFF. The second run of MDFF was performed under the same setting, and the resulting model was used for deposition. The stereochemistry of the resulting models was validated using MolProbity. All figures were prepared using PyMOL.

### Validation of ACKR3·CXCL12_WT_ structures by disulfide cross-linking in *Sf9* cells

The protocol for receptor-chemokine disulfide cross-linking in *Sf9* cells was adapted from previously described methods (*22*). Cross-links were detected by a C-terminal HA tag on the chemokine and reported as a fraction of previously established positive controls (ACKR3-R197C^ECL2^·CXCL12-Y7C and CXCR4-D187C·vMIP-II-W5C). Briefly, single cysteines were introduced into ACKR3 and CXCL12_WT_ (C-terminally tagged with an HA epitope) pfastBac constructs (described above) by QuikChange. Baculoviruses were then prepared for the ACKR3 and CXCL12 cysteine mutants as described above. The proteins were coexpressed by adding both receptor and chemokine baculoviruses, each at an MOI of 6, to 2.5 ml of *Sf9* cells at a density of 2.0 × 10^6^/ml in a 24–deep well block. The cells were incubated at 27°C with shaking at 300 rpm for 48 hours, after which 90 μl of cells was transferred to Protein LoBind tubes (Fisher Scientific) and 10 μl of 5 μM CXCL12_LRHQ_ was added to displace any noncovalently bound CXCL12_WT_. After incubating for 2 hours at room temperature, 10 μl of cells was transferred to a 96-well assay plate and incubated with 10 μl of anti-HA phycoerythrin-conjugated antibody (Miltenyi Biotec, 120-002-687) [diluted 1:100 in tris-buffered saline (TBS) + 4% (w/v) BSA] for 30 min at 4°C. Following incubation, 180 μl of TBS was added to each well and samples were analyzed using a Guava benchtop mini-flow cytometer (EMD Millipore). Data analysis was performed using FlowJo software version 10 (FlowJo LLC, Ashland, OR). The geometric mean fluorescence intensity (GMFI) for each cross-link was compared to a simultaneous experiment using an established efficient cross-linking construct, ACKR3-R197C^ECL2^·CXCL12-Y7C (*24*) as a positive control. Final reported values reflect the percentage of each GMFI compared to the ACKR3·CXCL12 control pair.

### BRET-based β-arrestin2 recruitment assay

Human β-arrestin2 recruitment was measured with a BRET2 assay as described previously (*14*). Briefly, HEK293T cells were seeded in six-well dishes at 750,000 cells per well in Dulbecco’s modified Eagle’s medium + 10% fetal bovine serum. The following day, each well was transfected transiently with 50 ng of Flag-ACKR3-RlucII and 1000 ng of GFP10–β-arrestin2 (a gift from N. Heveker, Université de Montréal) using TransIT transfection reagent (MirusBio, Madison, WI) as per the manufacturer’s protocol and cells were cultured for 2 days after transfection. For testing mutations in the orthosteric pocket and ACKR3-Y257^6.40^L, the N-terminal FLAG tag was omitted because it was observed to obfuscate the impact of mutations on constitutive recruitment.

To characterize the Y257^6.40^L mutation, cells expressing ACKR3 or ACKR3-Y257^6.40^L were washed with PBS buffer and resuspended in PBS buffer supplemented with 0.1% (w/v) glucose. A total of 100,000 cells were aliquoted into each well of a 96-well, white, clear-bottom plate (BD Falcon) and buffer was added to a final volume of 90 μl per well. Cells were incubated for 40 min at 37°C, 10 μl of serially diluted CXCL12 was added, and the plate was incubated for 20 min at 37°C. β-Arrestin2 expression was quantified from GFP10 fluorescence at 510 nm (400-nm excitation) on a SpectraMax M5 fluorescent plate reader (Molecular Devices). The bottom of the plate was covered, 5 μM Deep Blue C (coelenterazine-400a) (VWR) was added, and BRET was measured on a Victor X Light multilabel plate reader (PerkinElmer) by dividing GFP10 emission at 515 nm with RlucII emission at 410 nm. Following the experiment, total luminescence was measured to check receptor-RlucII expression.

For testing the effects of CID25 and mutations ACKR3-Y51^1.39^A, Y268^6.51^A, and Q301^7.39^A, cells were washed with PBS, resuspended in Tyrode’s buffer [25 mM Hepes (pH 7.5), 140 mM NaCl, 2.7 mM KCl, 12 mM NaHCO_3_, 5.6 mM glucose, 0.5 mM MgCl_2_, 0.37 mM NaH_2_PO_4_], and diluted to 1.25 × 10^6^/ml. Cells (80 μl) were aliquoted into a white, clear bottom, tissue culture 96-well plate (BD Falcon) and incubated for 45 min at 37°C. The GFP10–β-arrestin2 fluorescence levels were then measured with a SpectraMax 5M fluorescence plate reader (excitation, 485 nm; emission, 538 nm; cutoff, 530 nm). Next, Prolum purple (methoxy e-Coelenterazine, Nanolight Technology) was added to a final concentration of 5 μM alone or along with CID25. After incubating for 5 min, the total luminescence was measured for all wells using a VictorX Light multilabel plate reader (PerkinElmer) or Tecan Spark (Tecan). Next, CXCL12_WT_ or CXCL12_LRHQ_ was added at the indicated concentrations and the plate was measured at 410 and 515 nm for experiments on VictorX. For experiments using the Tecan Spark, emissions were collected using default BRET2 settings (blue emission, 360 to 440 nm; red emission, 505 to 575 nm). The BRET ratios (515 nm/410 nm) were calculated for three independent experiments and normalized to WT ACKR3 responses and merged. Titrations were fit to a sigmoidal dose-response model using SigmaPlot 11.0 (Systat Software Inc.). Significance was assessed using the extra sum-of-squares *F* test.

### BRET-based indirect β-arrestin2 recruitment to tag-less receptors

Recruitment to tag-less receptors was observed by enhanced bystander BRET2 between RlucII-tagged β-arrestin2 and plasma membrane–bound rGFP-CAAX as previously described (*64*). HEK293T cells were plated as described above to 750,000 cells per well and transfected the following day with 26 ng of RlucII-tagged β-arrestin2, 342 ng of rGFP-CAAX, and 178.1 ng of tag-less receptor or empty vector DNA, all in pcDNA3.1 expression vectors. After 48 hours, the cells were washed and replated in Tyrode’s buffer as above and adhered for 45 min at 37°C. Acceptor expression was checked using a SpectraMax 5M plate reader as above, and white backing tape was applied. Next, Prolum Purple (5 μM final concentration) was added to each well and the plate was incubated at 37°C for 2 min before 5 min of initial BRET2 background reads using a TECAN Spark luminescence reader (Tecan) and default BRET2 settings as above. CXCL12 was added to each well at a final concentration of 100 nM, and the signal was recorded for 20 min. Three independent time courses were averaged together and presented as a merged dataset. β-Arrestin2 recruitment was quantified by integrating the area under the curve after CXCL12 addition using GraphPad Prism version 9.2.0 (GraphPad Software Inc., San Diego, CA). Significance was assessed using one-way analysis of variance (ANOVA) and Dunnett’s multiple comparison test using SigmaPlot 11.0 (Systat Software Inc.). Surface receptor expression was quantified by labeling with R-phycoerythrin–conjugated anti-ACKR3 antibody (11G8, Fisher Scientific) and read using a GuavaCyte benchtop flow cytometer (Millipore). The GMFI for each sample was baseline-corrected to cells transfected with empty vector instead of ACKR3.

### BRET-based G protein activation assay

Activation of the heterotrimeric G proteins was observed with a BRET1 assay as previously described (*65*). HEK293T cells were plated in six-well dishes as described above at 750,000 cells per well. The next day, the cells were transfected using TransIT with 50 ng of Gα_i3_-RlucII, 500 ng of Venus(156-239)-Gβ_1_, 500 ng of Venus(1-155)-Gγ_2_ (a gift from N. Lambert, Augusta University), and 500 ng of receptor or empty vector DNA, all in pcDNA3.1 expression vectors. After 48 hours, the cells were washed and replated as above and allowed to re-adhere for 45 min at 37°C. After the Venus fluorescence was measured using a SpectraMax plate reader (same setup as the β-arrestin 2 assay) and the white tape was applied, 10 μM coelenterazine h (VWR) was added to each well and the initial basal BRET1 values were measured using a Tecan Spark luminescence reader (Tecan) using default BRET1 settings (blue emission, 430 to 480 nm; red emission, 505 to 590 nm) for 5 min. Next, chemokine or small molecules were added at final concentrations of 100 nM and 1 μM, respectively, and the BRET1 signal was tracked for an additional 10 min. Experiments were conducted three times in triplicate, and representative results are shown. Significance was assessed by one-way ANOVA followed by Dunnett’s multiple comparisons test.

### Scintillation proximity assay

ACKR3 was reconstituted into biotinylated MSP1E3D1 POPC/1-palmitoyl-2-oleoyl-sn-glycero-3-phospho-l-serine (POPS; Avanti) 3:2 molar ratio NDs as described above except that the step where Talon resin was added to remove empty NDs was omitted. NDs (200 fmol empty NDs or NDs containing ACKR3), Fabs (CID24 or CID25; final concentrations, 0, 0.2, or 1 μM), streptavidin-coated polyvinyltoluene scintillation proximity assay beads (132 μg; PerkinElmer), and buffer [200 mM NaCl, 5 mM CaCl_2_, 2 mM MgCl_2_, 60 mM Hepes (pH 7.5), 0.04% (w/v) BSA, and 3.98% (v/v) glycerol in a final reaction volume of 100 μl] were added to a white 96-well low-binding surface OptiPlate (PerkinElmer) in a total volume of 89 μl. The plate was shaken for 30 min at room temperature to allow immobilization of the NDs onto the beads. ^125^I-CXCL12 (2200 Ci/mmol; PerkinElmer) was diluted in 120 mM NaCl, 60 mM Hepes (pH 7.5), and 0.2% (w/v) BSA. Radioligand (10 μl) was added to the plate at a final concentration of 61 pM, and association was measured on a TopCount NXT device (PerkinElmer) in 1-min reads at 20°C. After reaching equilibrium, dissociation of ^125^I-CXCL12 was initiated by addition of 1 μl of 10 mM (final concentration, 100 μM) ACKR3 agonist VUF11207 (*66*) (Sigma-Aldrich) and followed in 1-min reads at 20°C. Duplicate measurements were averaged, and specific binding was determined by subtracting nonspecific binding to empty NDs from total binding to ACKR3-containing NDs. The decrease in specific binding as a function of time was fitted to a one-phase exponential decay model using GraphPad Prism version 9.2.0 (GraphPad Software Inc., San Diego, CA). Data from three independent experiments were averaged, and the statistical significance was assessed by one-way ANOVA followed by Dunnett’s multiple comparisons test.

## Supplementary Materials

### This PDF file includes:

Figs. S1 to S15 Tables S1 to S3 References
