# Supplementary material for: Structures of atypical chemokine receptor 3 reveal the basis for its promiscuity and signaling bias
Source: Sci Adv. 2022 Jul 13;8(28):eabn8063. doi: 10.1126/sciadv.abn8063 (PMC9278869; doi:10.1126/sciadv.abn8063)
Supplement: Supplementary file 1 — Figs. S1 to S15 Tables S1 to S3 References [file sciadv.abn8063_sm.pdf]

Science Advances
AAAS

Supplementary Materials for

Structures of atypical chemokine receptor 3 reveal the basis for its promiscuity and signaling bias

Yu-Chen Yen *et al.*

Corresponding author: John J. G. Tesmer, jtesmer@purdue.edu; Tracy M. Handel, thandel@health.ucsd.edu

Sci. Adv. **8**, eabn8063 (2022)
DOI: 10.1126/sciadv.abn8063

The PDF file includes:

Figs. S1 to S15
Tables S1 to S3
Legend for movie S1
References

Other Supplementary Material for this manuscript includes the following:

Movie S1

Table S1. Summary of determined structures

PDB entry	Complex (resolution)	cartoon	CID25 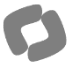	ACKR3 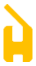	Ligand 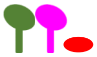	CID24 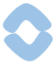	Anti-Fab NB 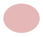
**7SK3**	NB-CID25-ACKR3·CXCL12$_{WT}$-CID24-NB (3.8 Å)	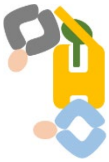	+	+	CXCL12$_{WT}$	+	+
**7SK4**	CID25-ACKR3·CXCL12$_{LRHQ}$-CID24 (3.3 Å)	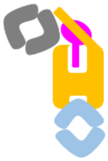	+	+	CXCL12$_{LRHQ}$	+	-
**7SK5**	ACKR3·CXCL12$_{WT}$-CID24-NB (4.0 Å)	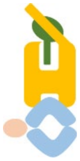	-	+	CXCL12$_{WT}$	+	+
**7SK6**	ACKR3·CXCL12$_{LRHQ}$-CID24 (4.0 Å)	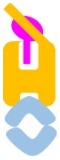	-	+	CXCL12$_{LRHQ}$	+	-
**7SK7**	NB-CID25-ACKR3·CXCL12$_{WT}$·CCX662 (3.3 Å)	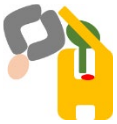	+	+	CXCL12$_{WT}$ & CCX662	-	+
**7SK8**	CID25-ACKR3·CXCL12$_{WT}$·CCX662-CID24 (3.3 Å)	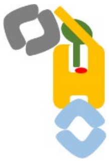	+	+	CXCL12$_{WT}$ & CCX662	+	-
**7SK9**	ACKR3·CCX662-CID24 (3.8 Å)	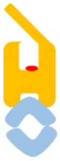	-	+	CCX662	+	-

Table S2. Cryo-EM data collection, refinement, and validation statistics

Sample	NB-CID25-ACKR3·CXCL12$_{WT}$-CID24-NB (EMDB-25171) (PDB 7SK3)	CID25-ACKR3·CXCL12$_{LRHQ}$-CID24 (EMDB-25172) (PDB 7SK4)	ACKR3·CXCL12$_{WT}$-CID24-NB (EMDB-25173) (PDB 7SK5)	ACKR3·CXCL12$_{LRHQ}$-CID24 (EMDB-25174) (PDB 7SK6)
**Data collection and processing**				
Magnification	81,000 X			
Voltage (kV)	300			
Electron exposure (e–/Å^2)	53.8			
Defocus range (μm)	1 ~ 3			
Pixel size (Å)	1.08			
Symmetry imposed	C1			
Initial particle images (no.)	4,117,503	4,376,522	4,117,503	5,203,100
Final particle images (no.)	528,797	317,314	397,340	322,105
Map resolution (Å)<br>FSC threshold 0.143	3.8	3.3	4.0	4.0
**Refinement**				
Map sharpening *B* factor (Å^2)	174	81	206	66
Map CC	0.82	0.79	0.81	0.52
Model composition<br>Non-hydrogen atoms<br>Protein residues<br>Ligands	 6803<br>831<br>CLR:8	 6656<br>831<br>CLR:2	 7232<br>897<br>CLR:7	 4689<br>585<br>0
B factors (Å^2)<br>Protein<br>Ligand	 43.2<br>35.1	 85.7<br>93.1	 90.1<br>62.8	 N/A*<br>N/A*
R.m.s. deviations<br>Bond lengths (Å)<br>Bond angles (°)	 0.003<br>0.442	 0.002<br>0.427	 0.002<br>0.429	 0.019<br>1.996
Validation<br>MolProbity score<br>Clashscore<br>Poor rotamers (%)	 1.61<br>5.05<br>0	 1.70<br>5.89<br>0	 1.69<br>6.42<br>0	 0.94<br>0.11<br>0.82
Ramachandran plot<br>Favored (%)<br>Allowed (%)<br>Disallowed (%)	 94.99<br>5.01<br>0	 94.49<br>5.51<br>0	 95.20<br>4.80<br>0	 94.05<br>4.20<br>1.75

* Model is fit with MDFF without further refinements.

Sample	NB-CID25-ACKR3·CXCL12$_{WT}$·CCX662 (EMDB-25175) (PDB 7SK7)	CID25-ACKR3·CXCL12$_{WT}$·CCX662-CID24 (EMDB-25176) (PDB 7SK8)	ACKR3·CCX662-CID24 (EMDB-25177) (PDB 7SK9)
**Data collection and processing**			
Magnification	81,000 X		
Voltage (kV)	300		
Electron exposure (e–/Å^2)	53.8		
Defocus range (μm)	1 ~ 3		
Pixel size (Å)	1.08		
Symmetry imposed	C1		
Initial particle images (no.)	3,385,553	4,370,553	4,370,553
Final particle images (no.)	814,168	605,556	297,347
Map resolution (Å)<br>FSC threshold 0.143	3.3	3.3	3.8
**Refinement**			
Map sharpening *B* factor (Å^2)	141	129	142
Map CC	0.80	0.83	0.82
Model composition<br>Non-hydrogen atoms<br>Protein residues<br>Ligands	 6805<br>854<br>CCX662:1<br>CLR:1	 6876<br>835<br>CCX662:1<br>CLR:6<br>LMN:1	 5700<br>716<br>CCX662:1<br>CLR:2
B factors (Å^2)<br>Protein<br>Ligand	 56.5<br>53.1	 35.1<br>41.0	 39.3<br>27.4
R.m.s. deviations<br>Bond lengths (Å)<br>Bond angles (°)	 0.004<br>0.498	 0.003<br>0.470	 0.003<br>0.525
Validation<br>MolProbity score<br>Clashscore<br>Poor rotamers (%)	 1.48<br>4.67<br>0	 1.41<br>2.62<br>0	 1.51<br>4.23<br>0
Ramachandran plot<br>Favored (%)<br>Allowed (%)<br>Disallowed (%)	 96.37<br>3.63<br>0	 94.78<br>5.22<br>0	 95.61<br>4.39<br>0

Table S3. Quantified disulfide crosslinking efficiency between CXCL12 and CXCR4 or ACKR3 data points presented in Fig. 3E and F. Each replicate was measured in triplicate and normalized to known efficient crosslinking constructs as described in Materials and Methods. Averaged values are presented in the heat maps in Fig. 3.

	Crosslinking efficiency by flow cytometry						
	**Receptor mutant**	**CXCL12 mutant**	**rep1**	**rep2**	**rep3**	**Avg**	**StDev**
CXCR4	G3C	I28C	16.5	17.7	25.1	19.8	4.7
	G3C	K27C	128	150	129	135	12.5
	G3C	L26C	18.9	20.3	26.8	22.0	4.2
	G3C	H25C	72.1	80.7	92.3	81.7	10.1
	I4C	I28C	16.6	18.6	26.8	20.7	5.4
	I4C	K27C	42.9	34.5	40.7	39.4	4.4
	I4C	L26C	20.9	12.8	34.3	22.7	10.9
	I4C	H25C	58.1	77.8	73.7	69.9	10.4
	S5C	I28C	12.5	14.1	21.0	15.9	4.5
	S5C	K27C	78.1	62.9	59.7	66.9	9.8
	S5C	L26C	14.9	10.3	24.2	16.5	7.1
	S5C	H25C	54.7	90.4	77.5	74.2	18.1
	I6C	I28C	10.6	13.4	18.0	14.0	3.7
	I6C	K27C	25.1	51.0	33.6	36.6	13.2
	I6C	L26C	13.1	17.6	19.3	16.7	3.2
	I6C	H25C	28.4	47.4	32.5	36.1	10.0
	Y7C	I28C	11.9	16.6	21.8	16.8	5.0
	Y7C	K27C	59.2	72.9	55.5	62.5	9.2
	Y7C	L26C	13.6	20.8	23.1	19.2	5.0
	Y7C	H25C	77.4	92.9	93.6	88.0	9.2
	S9C	I28C	10.4	18.8	25.0	18.1	7.3
	S9C	K27C	46.1	86.4	59.8	64.1	20.5
	S9C	L26C	14.4	10.1	21.2	15.2	5.6
	S9C	H25C	72.7	98.2	72.8	81.2	14.7
ACKR3	T31C	I28C	52.1	32.2	51.8	45.4	11.4
	T31C	K27C	16.8	9.90	15.5	14.1	3.6
	T31C	L26C	29.0	17.3	25.4	23.9	6.0
	T31C	H25C	19.2	13.5	17.1	16.6	2.9
	T31C	S16C	16.5	9.90	17.1	14.5	4.0

	T31C	F13C	18.8	13.4	15.4	15.9	2.7
	D30C	I28C	9.80	15.0	12.3	12.4	2.6
	D30C	K27C	6.50	8.50	9.40	8.10	1.5
	D30C	L26C	6.6	8.5	2.2	5.8	3.3
	D30C	H25C	11.5	13.2	11.4	12.0	1.0
	D30C	S16C	5.8	8.0	6.4	6.7	1.2
	D30C	F13C	7.8	6.4	5.4	6.5	1.2
	V29C	I28C	98.9	64.0	73.1	78.7	18.1
	V29C	K27C	28.7	16.2	26.3	23.7	6.6
	V29C	L26C	51.3	29.2	35.4	38.6	11.4
	V29C	H25C	48.4	40.6	29.9	39.6	9.3
	V29C	S16C	16.5	13.2	10.8	13.5	2.9
	V29C	F13C	38.8	33.3	31.2	34.4	3.9

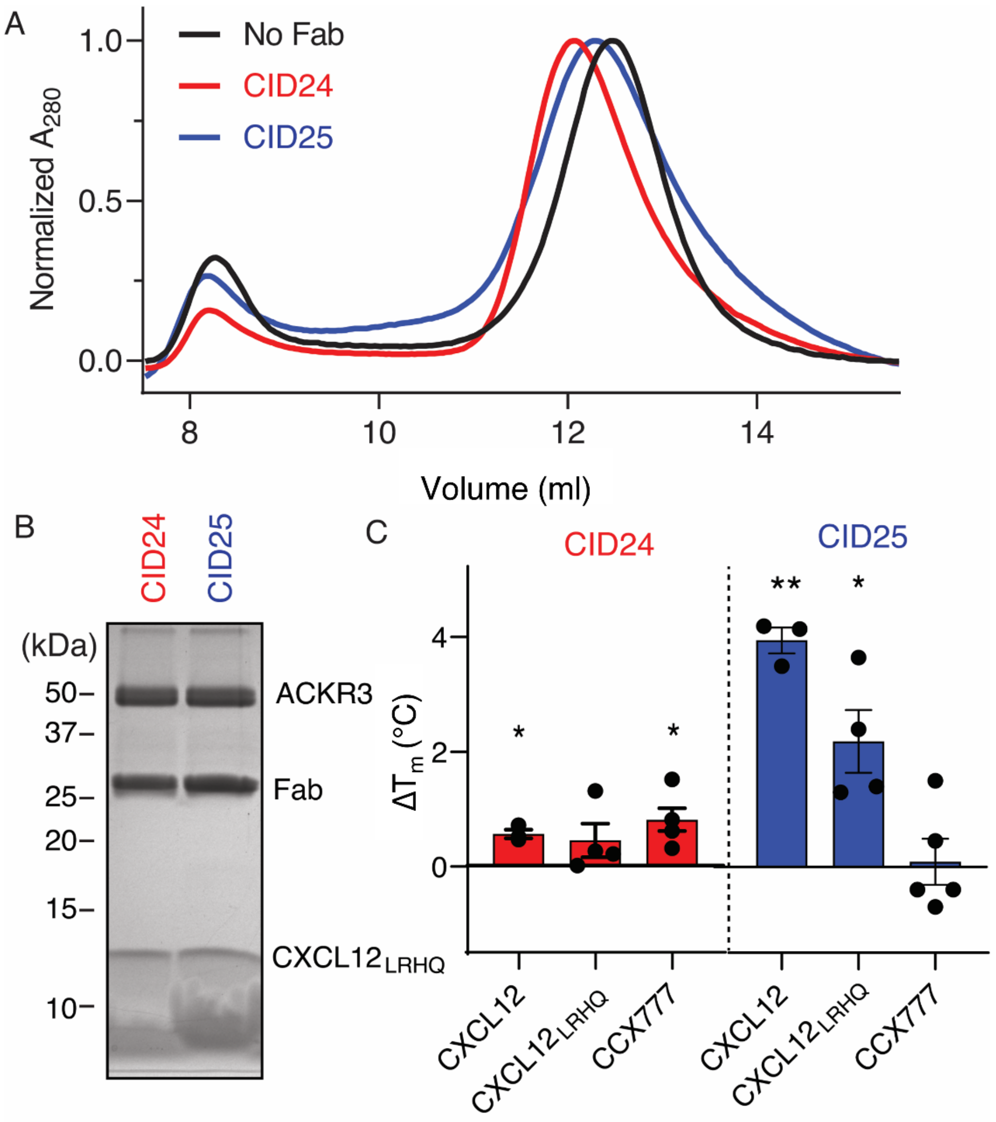

Figure S1. Characterization of ACKR3–Fab complexes. (A) The ACKR3·$CXCL12_{LRHQ}$ complex eluted at 12.5 ml when applied to a Superdex 200 10/300 column. Upon incubation with CID24 or CID25, the peak shifted to 12.1 and 12.3 ml, respectively, indicating formation of an ACKR3·$CXCL12_{LRHQ}$-Fab complex. (B) Peaks corresponding to ACKR3·$CXCL12_{LRHQ}$-Fab complexes (total volume 2 ml) were concentrated and analyzed by SDS-PAGE. Bands corresponding to ACKR3, Fab, and $CXCL12_{LRHQ}$ likewise indicated the presence of a ternary complex. (C) Effect of Fabs on the T_m of ACKR3 complex with $CXCL12_{WT}$, $CXCL12_{LRHQ}$, and the small molecule partial agonist CCX777 determined from CPM thermostability assays quantified as ΔT_m (T_m of ACKR3·ligand-Fab – T_m of ACKR3·ligand). Significance of the changed T_m values relative to the complex without Fab were determined from one-way repeated measure analysis of variance (ANOVA) with Dunnett's multiple comparison test (*, $P < 0.05$; **, $P < 0.01$; ***, $P < 0.001$), n=3, n=4 and n=5 for CXCL12, $CXCL12_{LRHQ}$, and CCX777, respectively.

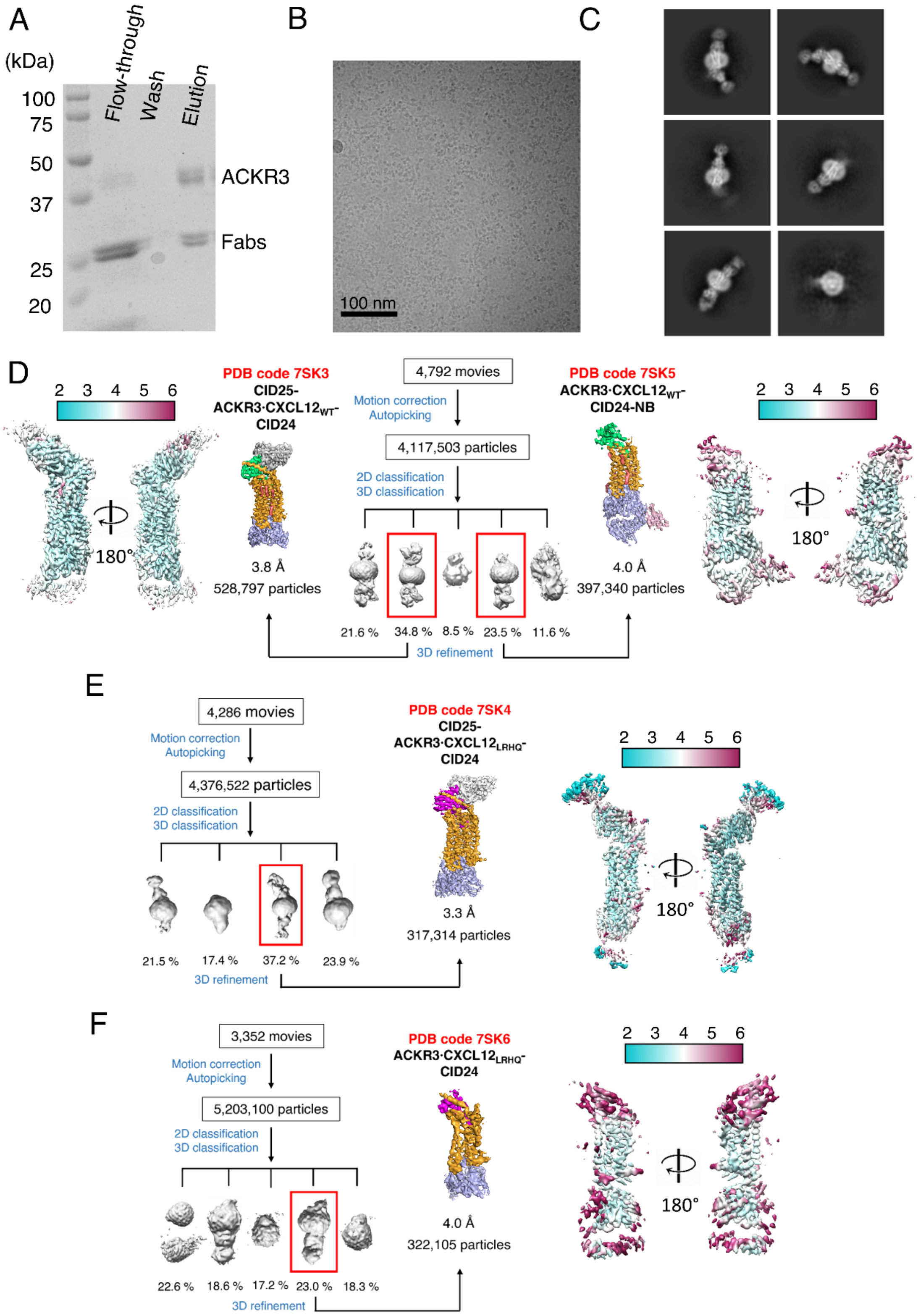
A
(kDa)
100
75
50
37
25
20
Flow-through
Wash
Elution
ACKR3
Fabs
B
100 nm
C
D
2 3 4 5 6
PDB code 7SK3
CID25-ACKR3·CXCL12WT-CID24
180°
3.8 Å
528,797 particles
4,792 movies
Motion correction
Autopicking
4,117,503 particles
2D classification
3D classification
21.6 %
34.8 %
8.5 %
23.5 %
11.6 %
3D refinement
PDB code 7SK5
ACKR3·CXCL12WT-CID24-NB
4.0 Å
397,340 particles
E
4,286 movies
4,376,522 particles
21.5 %
17.4 %
37.2 %
23.9 %
PDB code 7SK4
CID25-ACKR3·CXCL12LRHQ-CID24
3.3 Å
317,314 particles
F
3,352 movies
5,203,100 particles
22.6 %
18.6 %
17.2 %
23.0 %
18.3 %
PDB code 7SK6
ACKR3·CXCL12LRHQ-CID24
4.0 Å
322,105 particles

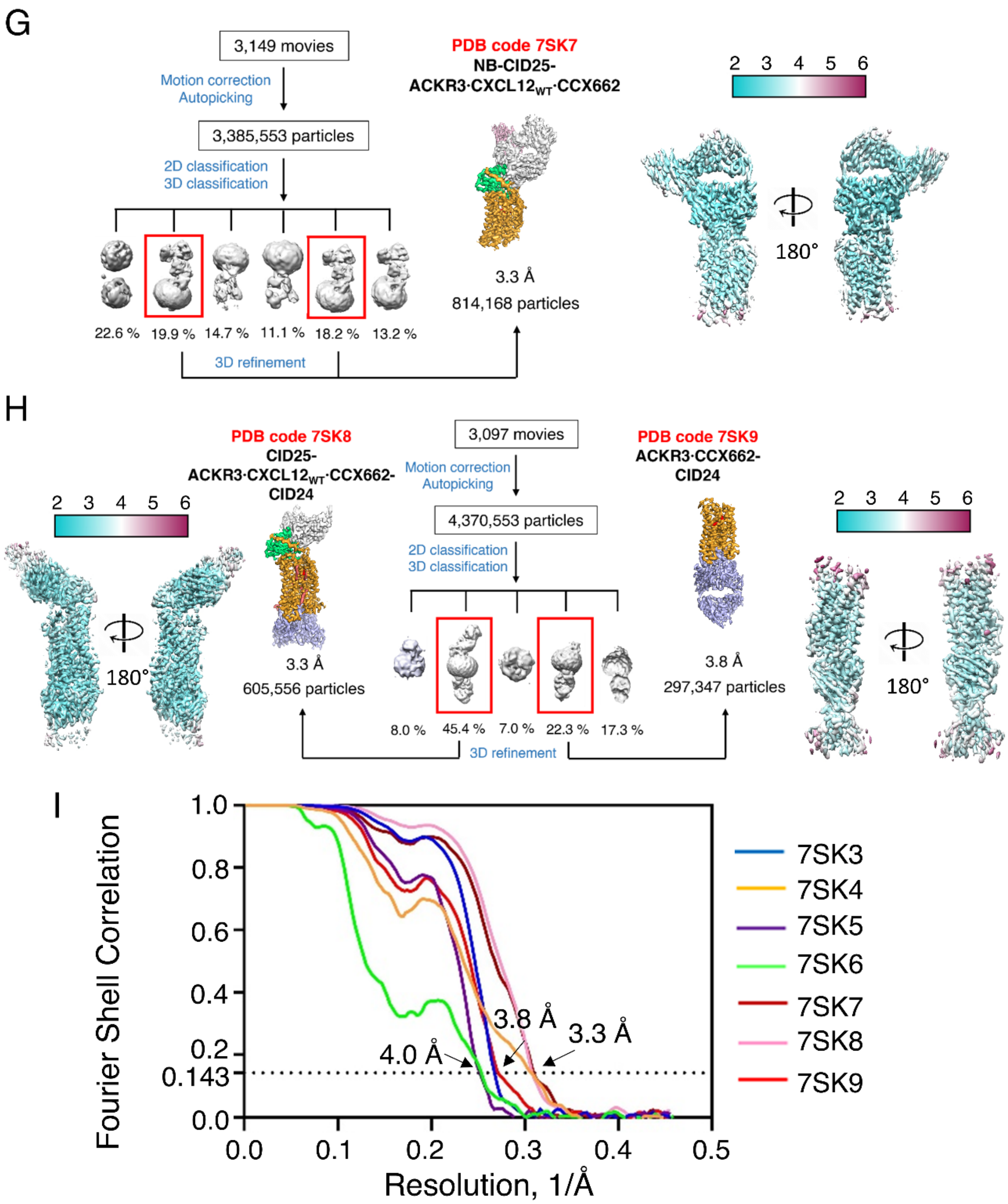

Figure S2. Sample preparation, cryo-EM data processing workflow, and resolution analysis of ACKR3 complexes. (A) Representative Coomassie blue stained gel of a ACKR3·CXCL12$_{WT}$-Fab complex isolated via an anti-FLAG M2 antibody. (B) Representative micrograph showing well-distributed complexes in vitrified ice. (C) Representative 2D class averages (box size = 324 Å, mask = 180 Å) with interpretable density for the micelle-bound ACKR3 and two Fabs. (D-H) Cryo-EM data processing workflow and local resolution map for each determined complex. (I) Fourier shell correlation (FSC) curves calculated from two independent reconstructions by cryoSPARC for each determined complex. The 0.143 cutoff is depicted as a dotted black line and defines the nominal resolution of the resulting maps.

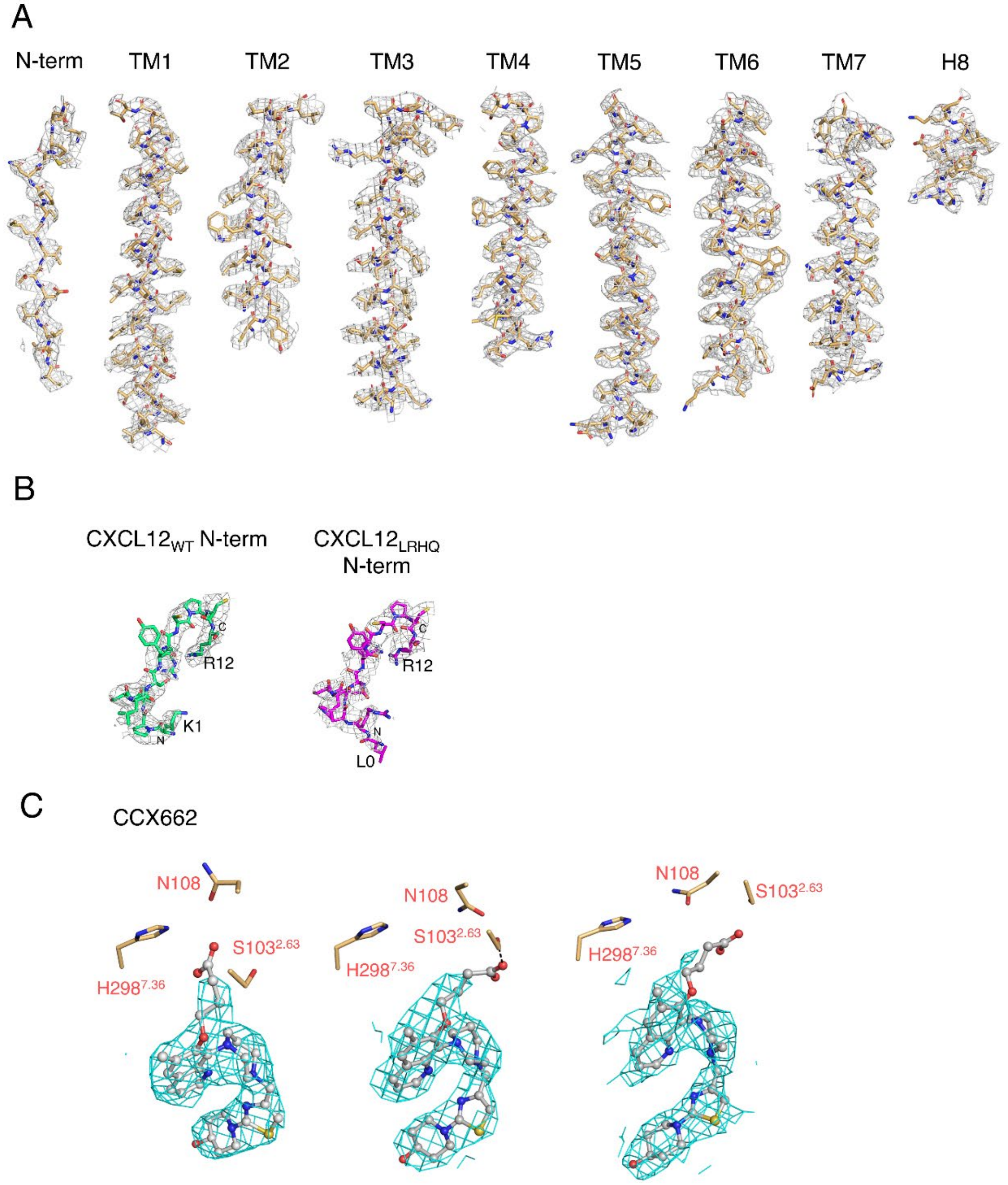

Figure S3. Cryo-EM map quality. (A) Density maps and models for the N-terminus, TM helices and H8 of ACKR3 in the CID25-ACKR3·CXCL12$_{WT}$-CID24 complex (PDB entry 7SK3). (B) Density maps and models for the CXCL12$_{WT}$ N-terminus (CID25-ACKR3·CXCL12$_{WT}$-CID24, PDB entry 7SK3), CXCL12$_{LRHQ}$ N-terminus (CID25-ACKR3·CXCL12$_{LRHQ}$-CID24 complex, PDB entry 7SK4). (C) Density maps and models for the ligand CCX662 (left: ACKR3·CCX662-CID24, PDB entry 7SK9; middle: CID25-ACKR3·CXCL12$_{WT}$·CCX662-CID24, PDB entry 7SK8; right: CID25- ACKR3·CXCL12$_{WT}$·CCX662, PDB entry 7SK7). ACKR3 residues within hydrogen bond range of the carboxylic acid moiety of CCX662 are shown as sticks and colored based on atom type. Black dashed lines indicate putative hydrogen bonds. The carboxylate moiety is poorly ordered and has a number of residues with which it could favorably interact.

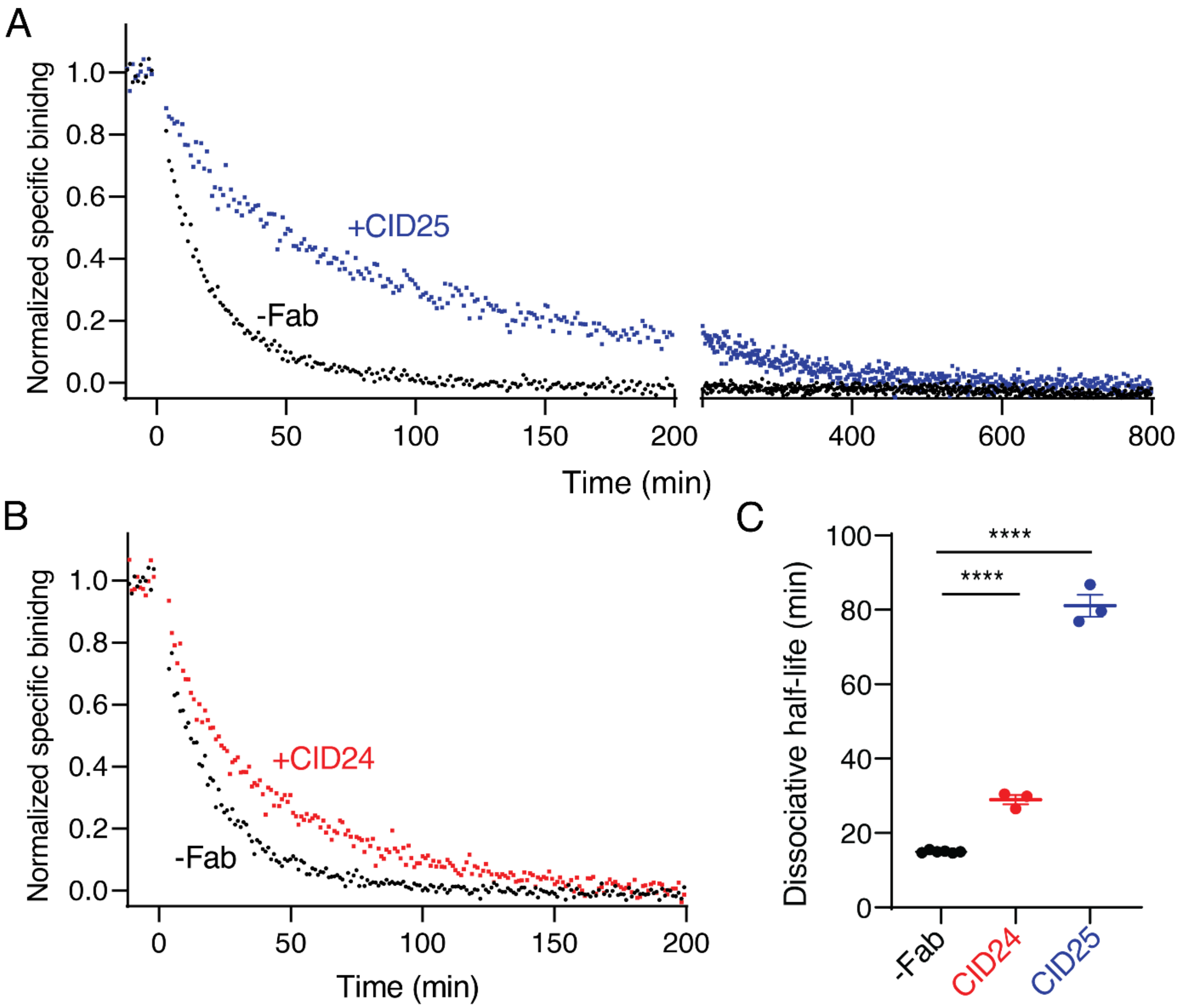

Figure S4. Fabs stabilize the ACKR3·CXCL12 complex. (A,B) Dissociation of [I^{125}]-CXCL12 (2200 Ci/mmol; Perkin Elmer) from ACKR3 in nanodiscs (NDs) measured as a decrease in specific binding in a scintillation proximity assay (*67*). ACKR3 was incubated with CXCL12 in the presence or absence of the indicated Fab until binding reached saturation. Dissociation was initiated by addition of a large excess of the small molecule agonist VUF11207 at t=0. Duplicate measurements were averaged and binding of [I^{125}]-CXCL12 to empty NDs was subtracted to obtain the specific binding. (C) Dissociation half-life of the ACKR3·CXCL12 complex determined from fitting of dissociation curves to single exponentials in GraphPad Prism version 9.2.0 (GraphPad Software, Inc., San Diego, CA). CID24 and CID25 significantly increase the dissociation half-life of CXCL12 as determined from a one-way analysis of variance (ANOVA) of three independent experiment with Dunnett's multiple comparison test (****, $P < 0.0001$).

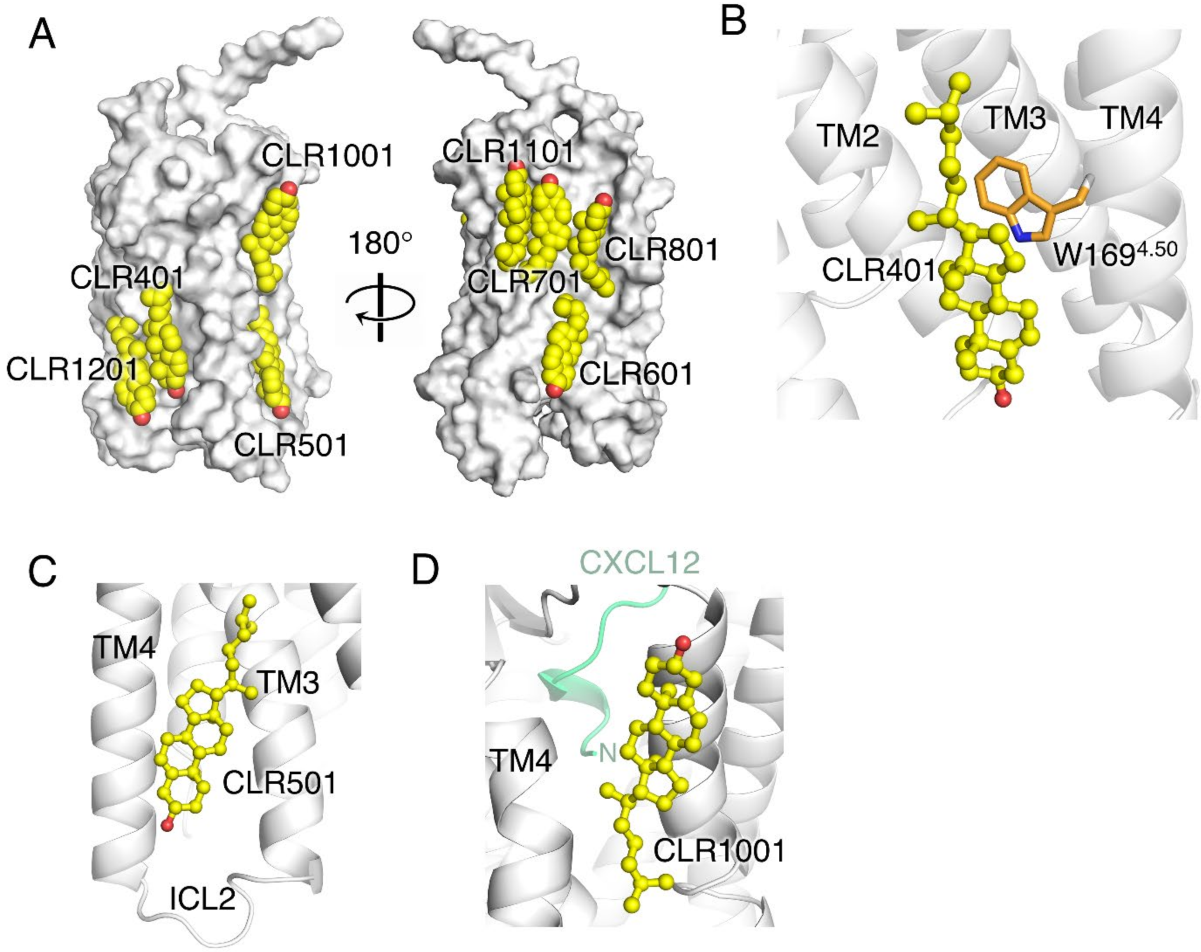

Figure S5. Cholesteryl hemisuccinate binding sites in ACKR3. Because the succinyl moieties were not ordered, they were modeled as cholesterol shown here with yellow carbons and red oxygens. (A) At least eight cholesterol binding sites were observed in ACKR3 (CID25-ACKR3·CXCL12$_{WT}$-CID24, PDB entry 7SK3), shown as a grey surface. There was evidence in the density maps for additional sites. (B) In the most conserved cholesterol binding site among the ACKR3 structures, the cholesterol moiety (CLR401) packs against the side chain W169$^{4.50}$ (orange carbons). (C) The second most conserved cholesterol binding site (CLR501) is close to ICL2. (D) Another common cholesterol site (CLR1001) was observed in three structures near the orthosteric binding pocket (CXCL12 N-terminus shown in green).

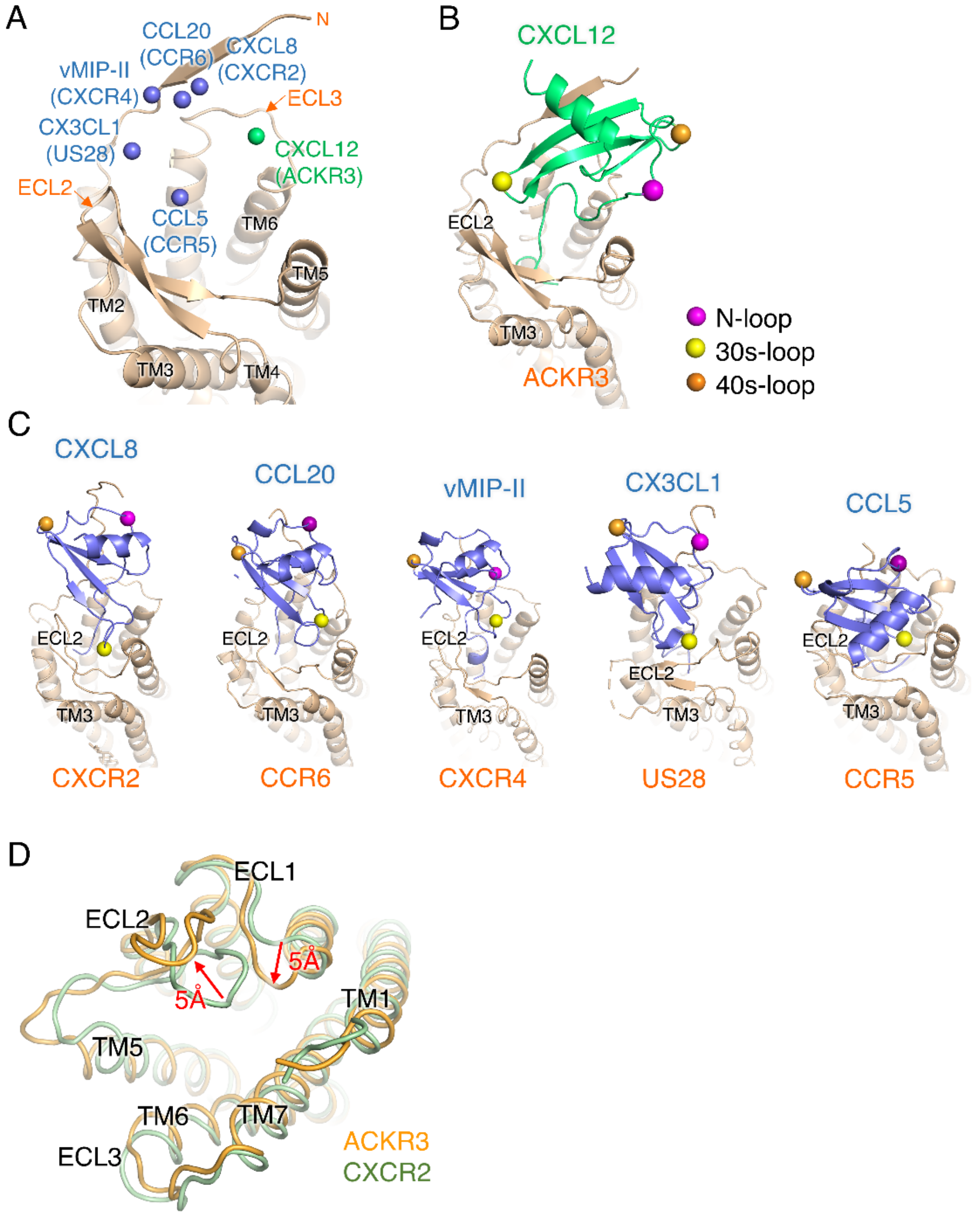

Figure S6. Survey of chemokine binding modes at their cognate receptors. (A) Center of mass of chemokines on the receptor. CXCR2 (PDB entry 6LFO: CXCR2 in complex with CXCL8 and $G\alpha_iG\beta_1\gamma_2$), CCR6 (PDB entry 6WWZ: CCR6 in complex with CCL20 and $G\alpha_oG\beta_1\gamma_2$), CXCR4 (PDB entry 4RWS: CXCR4 in complex with a viral chemokine antagonist), US28 (PDB entry 4XT1: US28 in complex with CX3CL1) and CCR5 (PDB entry 7F1R: CCR5 in complex with CCL5 and $G\alpha_iG\beta_1\gamma_2$) were first superimposed on ACKR3 (PDB entry 7SK3: CID25-ACKR3·CXCL12$_{WT}$-CID24) and their center of mass for bound chemokine determined using PyMOL (shown as blue and green spheres). (B) Cartoon representation of chemokine binding mode of ACKR3·CXCL12 and (C) of other chemokine-receptor structures from (A). (D) Extracellular view of ACKR3 (PDB entry 7SK3: CID25-ACKR3·CXCL12$_{WT}$-CID24) compared with CXCR2 in complex with CXCL8 and $G\alpha_iG\beta_1\gamma_2$ (PDB entry 6LFO). Arrows indicate differences in the conformation of ECLs in ACKR3 relative to the other structures.

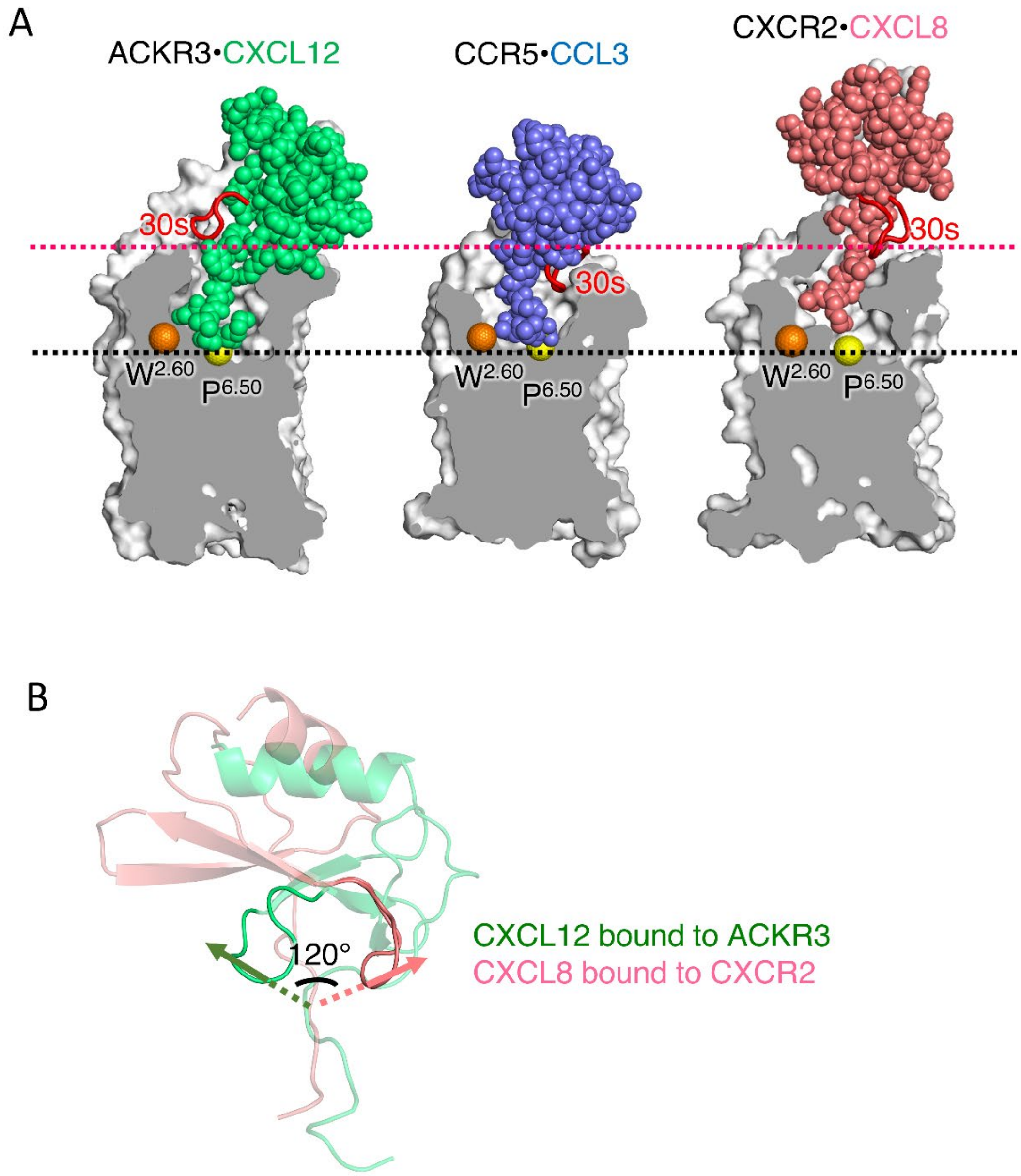

Figure S7. Comparison of chemokine binding modes of ACKR3 in complex with CXCL12 (PDB entry 7SK3: CID25-ACKR3·CXCL12$_{WT}$-CID24), with CCR5 in complex with CCL3 and $G\alpha_i G\beta_1\gamma_2$ (PDB entry 7F1Q) and CXCR2 in complex with CXCL8 (PDB entry 6LFO). (A) Sectioned view to describe the depth of binding in the orthosteric pocket. Chemokines are depicted with spheres and the 30s loop is shown as a red cartoon. The Cα atoms of conserved residues $W^{2.60}$ and $P^{6.50}$ are shown as spheres for receptor height alignment. The depth of N-terminus and 30s loop are indicated with black and red dash lines, respectively. (B) Comparison of the binding orientation of the 30s loop in the chemokines from the ACKR3 and CXCR8 complexes described in (A). The rotation between chemokines is defined by the angle between the vector between the Cα atoms of residues 30 and 31 of CXCL12 and CXCL8 (green and orange arrows, respectively).

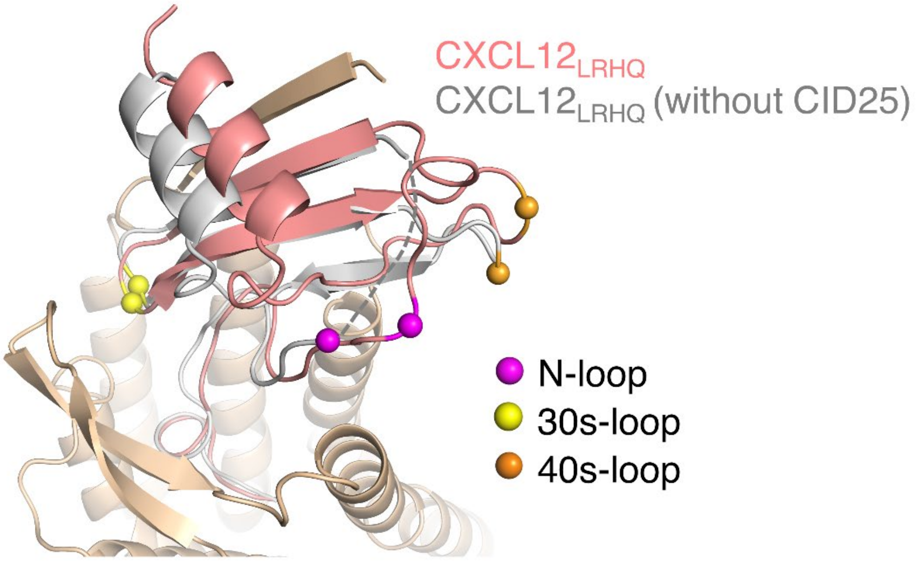

Figure S8. Superposition of receptors in the CID25-ACKR3·CXCL12$_{LRHQ}$-CID24 (PDB entry 7SK4) and ACKR3·CXCL12$_{LRHQ}$-CID24 (PDB entry 7SK6) complexes indicate the same binding mode for the bound chemokine in the absence of the Fab. The dashed line indicates a disordered loop in the latter model due to the absence of CID25.

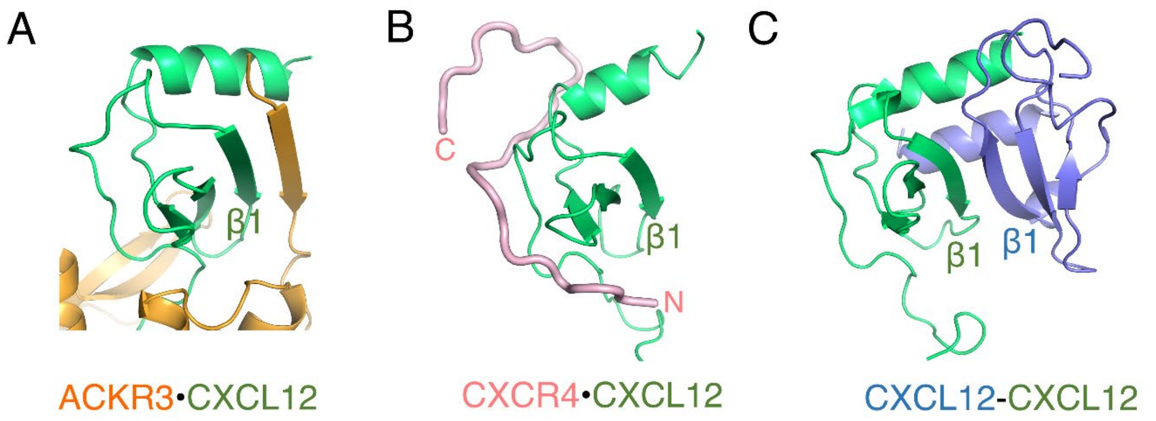

Figure S9. Binding of CXCL12 dimers is more readily accommodated by CXCR4 than by ACKR3. (A) In the structure of CID25-ACKR3·CXCL12$_{WT}$-CID24 (PDB entry 7SK3), the N-terminus of ACKR3 (orange) interacts with the β1 strand of CXCL12 (green). (B) NMR structure of CXCL12 in complex with the N-terminus of CXCR4 (PDB entry 2K04). (C) In the crystal structure of recombinant human CXCL12 (PDB entry 2J7Z), the chemokine forms a dimer via its β1 strand.

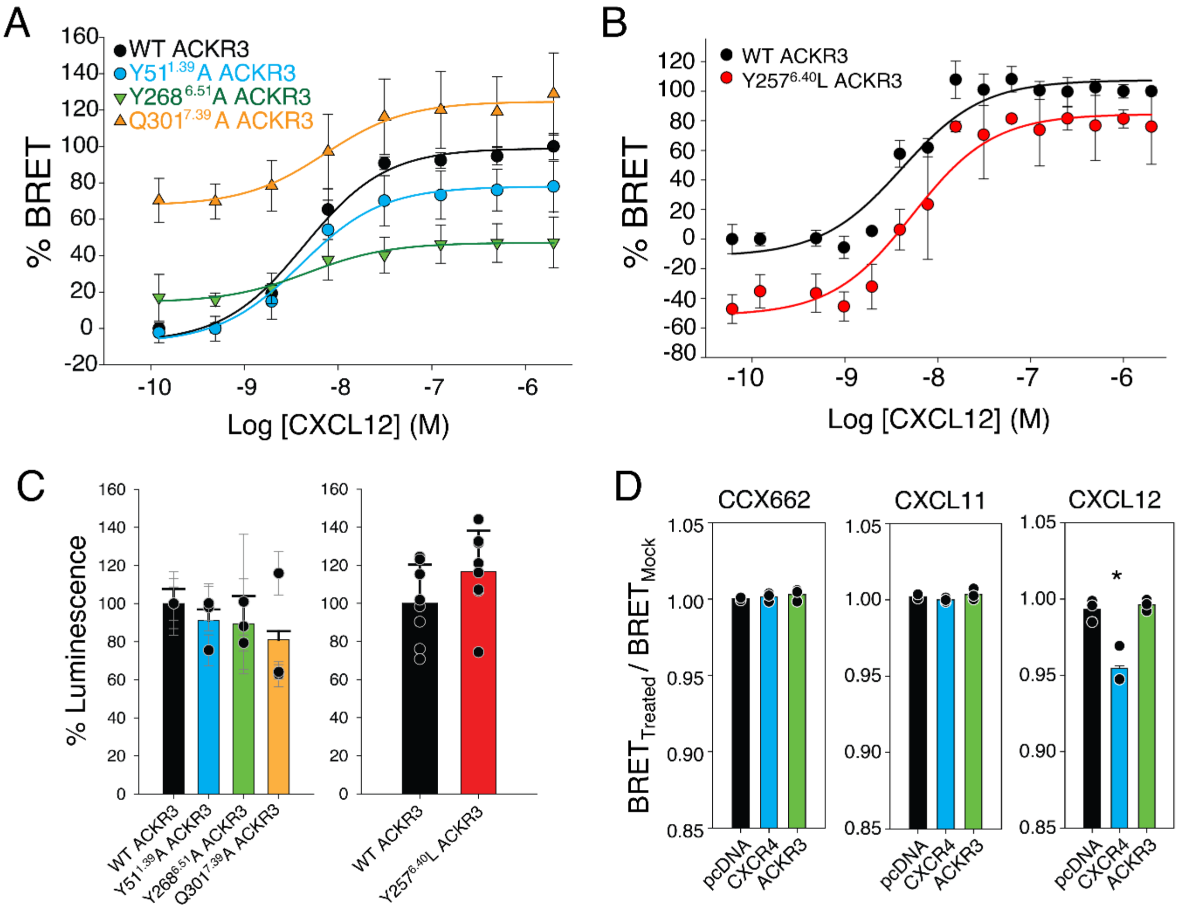

Figure S10. BRET analysis of arrestin binding and heterotrimeric G protein coupling. (A) Mutations to ACKR3 in the CXCL12 binding pocket alter chemokine efficacy and constitutive activity towards recruitment of β-arrestin2. Binding of GFP10-β-arrestin2 to ACKR3-RlucII was detected by BRET across a titration of CXCL12 concentrations and normalized as a percent of WT ACKR3 BRET. Q301A both showed enhanced constitutive interactions between ACKR3 and β-arrestin2 as evidenced by higher baselines (E_{min}, $P<0.001$). Y268A was also much less responsive to CXCL12. Y51 packs between TM2 and TM7 but makes no contact with ligands. Its mutation only subtly lowered efficacy (E_{max}, $P<0.001$). Curves are a composite of three independent experiments measured in triplicate with errors reported as standard deviations and statistics assessed with extra sum-of-squares F test. (B) Y257L reduces the constitutive activity of ACKR3. Recruitment of GFP10-β-arrestin2 to ACKR3-RlucII was diminished across the full titration curve for Y257L compared to WT, yet the efficacy is similar (E_{max} and E_{min}, $P<0.001$). Curves are composites of four separate experiments measured in triplicate and normalized to the WT ACKR3 signal with errors reported as standard deviations and statistics assessed by extra sum-of-squares F test. (C) Full spectrum RlucII luminescence for the constructs in (A) and (B) normalized to WT levels as a measure of expression. No construct was significantly different compared to WT ACKR3-RlucII. (D) β-arrestin agonists for ACKR3 (CCX662, CXCL11, and CXCL12) do not activate G proteins. G protein activation was measured by BRET decrease due to dissociation of $G\alpha_i$-RlucII and Gβγ-Venus. No ligands tested led to a drop in BRET except for CXCL12 with CXCR4. Three independent experiments performed in triplicate were combined. Significance (*, $P<0.05$) was determined by one-way ANOVA and Dunnett's Multiple Comparison Test. All errors are reported as standard deviations.

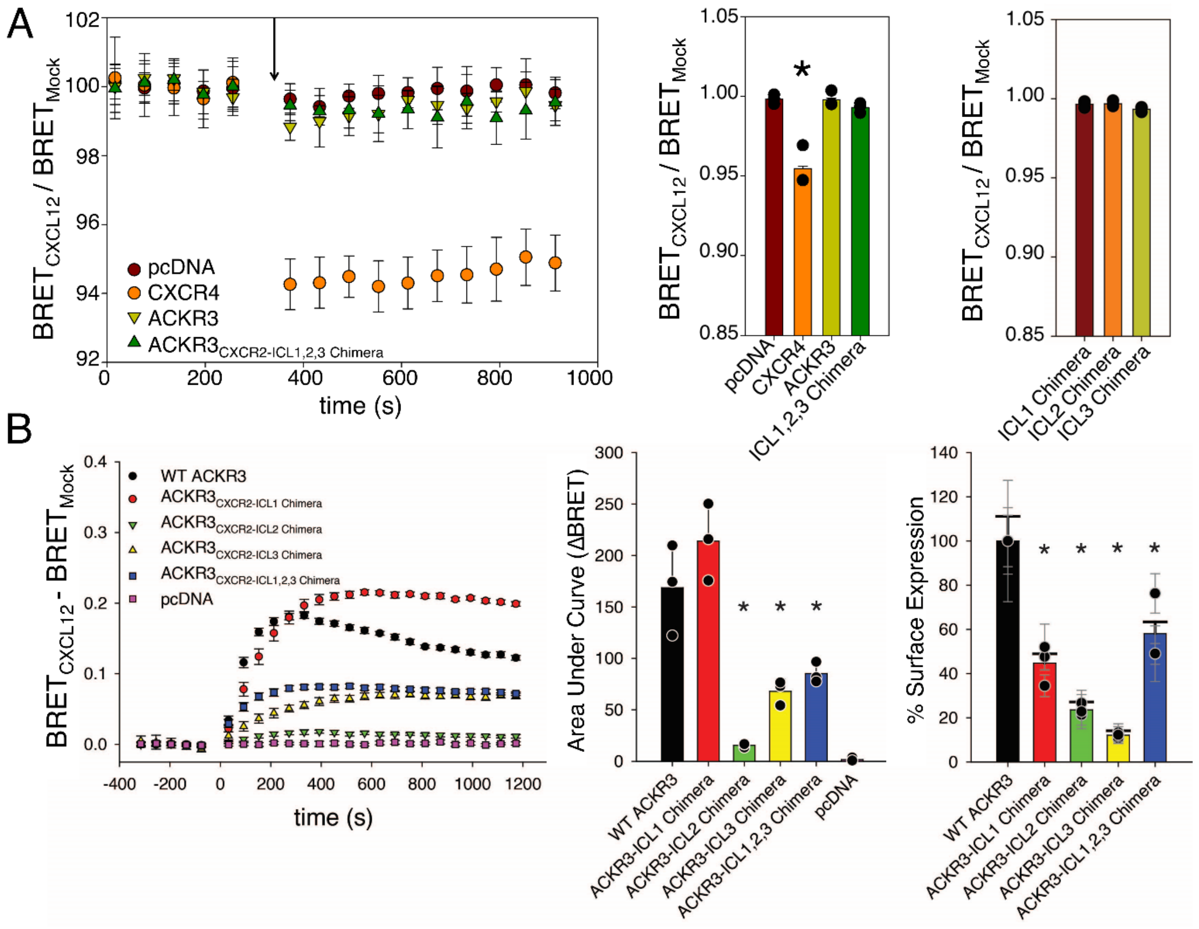

Figure S11. G protein activation by ACKR3 chimeras. (A) Left: G protein activation detected by a decrease in the BRET signal between Gαᵢ-RlucII and Gβγ-Venus due to dissociation of the heterotrimer confirms that ACKR3 (yellow) shows no CXCL12 (added at the arrow) induced activation of G protein over the empty vector control, pcDNA (dark red). CXCR4 shows a robust decrease in BRET with CXCL12 treatment (orange), indicative of G protein activation. Representative traces are presented and errors are reported as standard deviation. Right: ACKR3-chimeras generated by replacing the ICLs of ACKR3 with those of CXCR2 did not activate G proteins, but loss of β-arrestin2 recruitment (shown in B) suggests issues with expression, folding, or targeting of the chimeras. The change in BRET is quantified across three independent experiments done in triplicate. Significance (*, P<0.05) was determined by one-way ANOVA and Dunnett's Multiple Comparison Test. (B) Binding of RlucII-β-arrestin2 to CXCL12 activated ACKR3 was detected by BRET to a bystander acceptor (rGFP-CAAX) on the plasma membrane. The data shows impairment by all chimeras except ACKR3-ICL1 (area under the curve, P<0.05). Time courses are the composite average of three independent experiments done in triplicate; error bars are standard deviation. Surface expression of each ACKR3 mutant was determined by fluorescent antibody labeling and flow cytometry. Swapping the ICLs of CXCR2 into ACKR3 all had a negative effect on surface presentation of the receptor. Areas under the curves were calculated using GraphPad Prism version 9.2.0 (GraphPad Software, Inc., San Diego, CA) and statistical significance was determined by one-way ANOVA followed by Dunnett's Multiple Comparison Test.

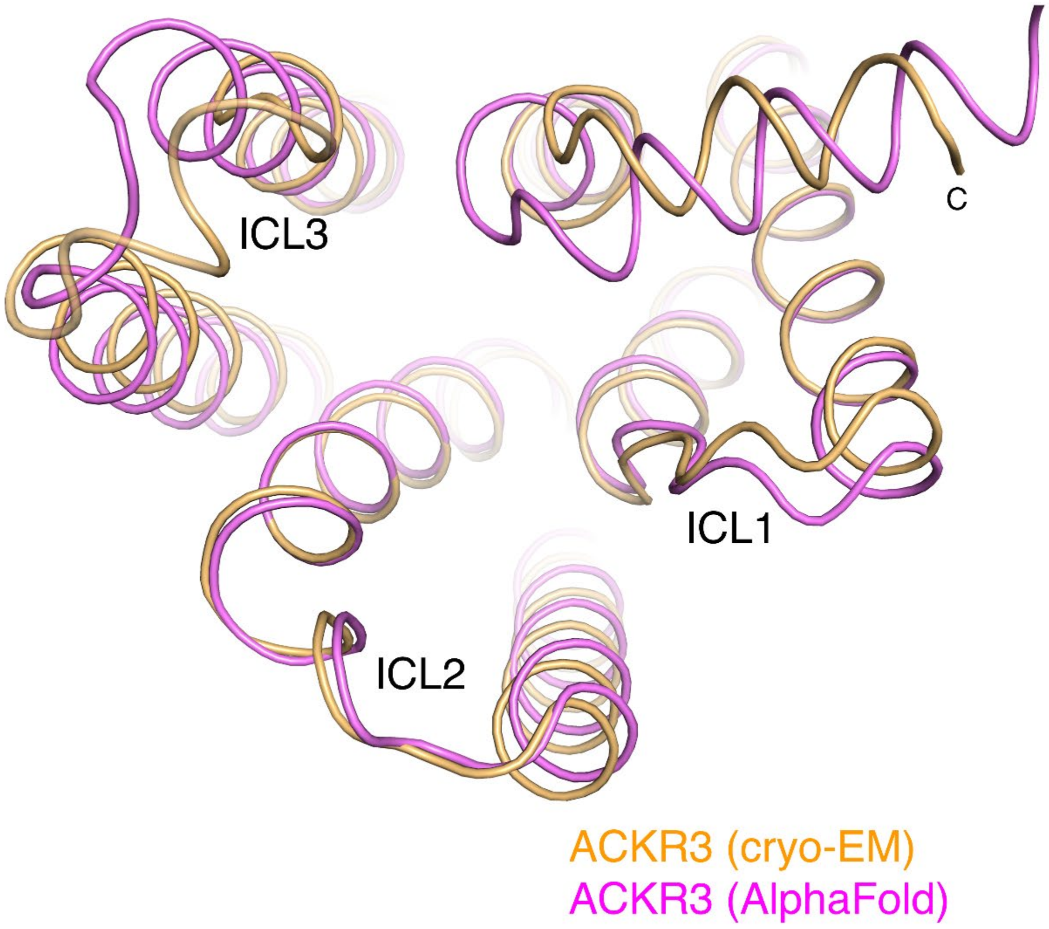

Figure S12. Intracellular view of ACKR3 (PDB entry 7SK3) determined with cryo-EM (orange) superimposed with the structure predicted by AlphaFold2 (pink) (*38*).

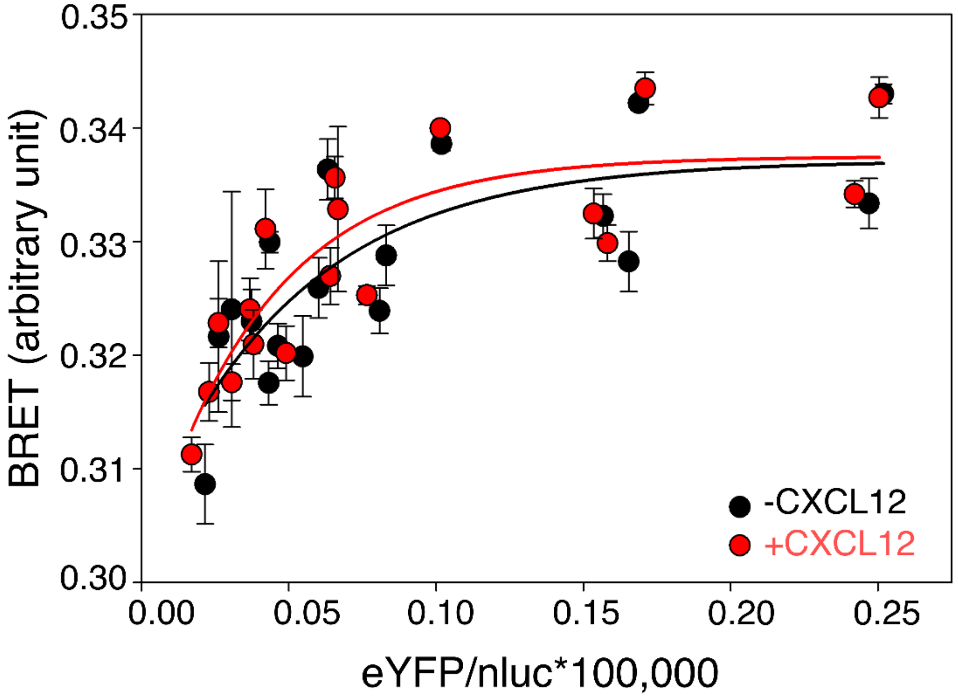

Figure S13. ACKR3 is closely associated with G proteins in cells. BRET between Gα_i-nluc and ACKR3-eYFP shows a hyperbolic increase with increasing expression of the receptor. The interaction was nearly identical in the presence (red) or absence (black) of CXCL12, suggesting the lack of a functional interaction.

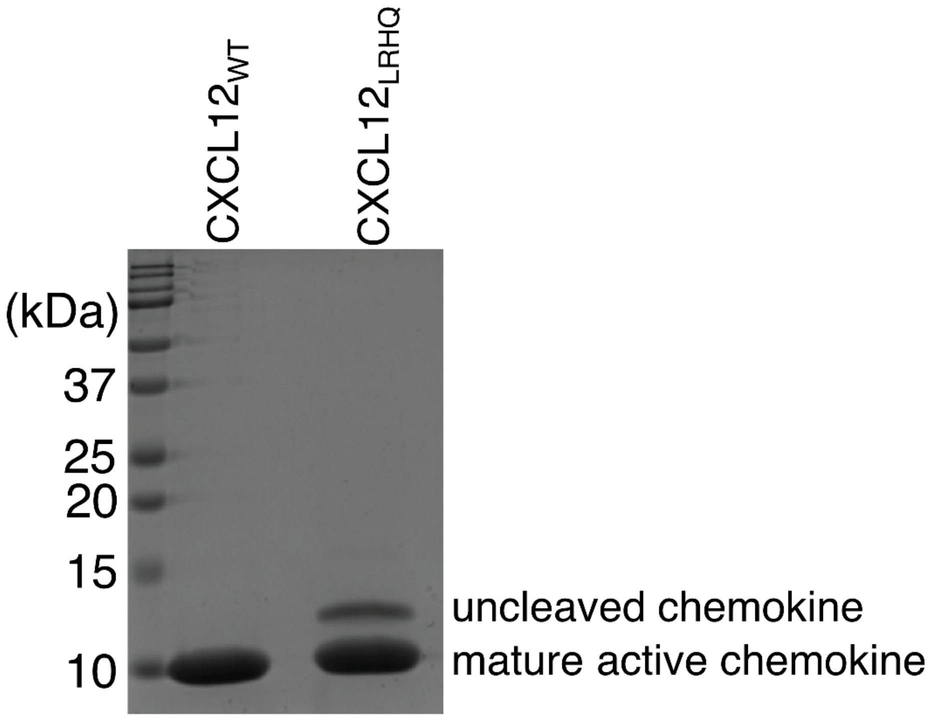

Figure S14. The purity of chemokines expressed in *E. coli* was assessed by SDS-PAGE followed by Coomassie staining. The dominant band at about 10 kDa represents the mature active chemokine. A small fraction of the $CXCL12_{LRHQ}$ material, represented by the fainter higher molecular weight band, was uncleaved and not removed by HPLC. However, the presence of the uncleaved material in the $CXCL12_{LRHQ}$ preparation does not impact conclusions because it was only used in the experiment to determine the effect of +/- CID25 on arrestin recruitment; in this experiment the same amount of active cleaved chemokine was added to both samples. The uncleaved material is unlikely to bind ACKR3.

CID24

>Light Chain

SDIQMTQSPSSLSASVGDRVTITCRASQSVSSAVAWYQQKPGKAPKLLIYSASSLYSGVPSR
FSGSRSGTDFTLTISSLQPEDFATYYCQQSYYYPITFGQGTKVEIKRTVAAPSVFIFPPSDSQL
KSGTASVVCLLNNFYPREAKVQWKVDNALQSGNSQESVTEQDSKDSTYSLSSTLTLSKADY
EKHKVYACEVTHQGLSSPVTKSFNRGEC

>Heavy Chain

EISEVQLVESGGGLVQPGGSLRLSCAASGFNISSSSIHWVRQAPGKGLEWVASISPSYGYTS
YADSVKGRFTISADTSKNTAYLQMNSLRAEDTAVYYCARVSYWDWTWGWSKYEGMDYWG
QGTLVTVSSASTKGPSVFPLAPSSKSTSGGTAALGCLVKDYFPEPVTVSWNSGALTSGVHT
FPAVLQSSGLYSLSSVVTVPSSSLGTQTYICNVNHKPSNTKVDKKVEPKSCDKTHT

CID25

>Light Chain

SDIQMTQSPSSLSASVGDRVTITCRASQSVSSAVAWYQQKPGKAPKLLIYSASSLYSGVPSR
FSGSRSGTDFTLTISSLQPEDFATYYCQQYYYPLFTFGQGTKVEIKRTVAAPSVFIFPPSDSQ
LKSGTASVVCLLNNFYPREAKVQWKVDNALQSGNSQESVTEQDSKDSTYSLSSTLTLSKAD
YEKHKVYACEVTHQGLSSPVTKSFNRGEC

>Heavy Chain

EISEVQLVESGGGLVQPGGSLRLSCAASGFNFSYSSIHWVRQAPGKGLEWVAYIYSSYGYT
SYADSVKGRFTISADTSKNTAYLQMNSLRAEDTAVYYCARVYPWWYYKYYHGALDYWGQG
TLVTVSSASTKGPSVFPLAPSSKSTSGGTAALGCLVKDYFPEPVTVSWNSGALTSGVHTFPA
VLQSSGLYSLSSVVTVPSSSLGTQTYICNVNHKPSNTKVDKKVEPKSCDKTHT

Figure S15. Sequences of the polypeptides composing Fabs CID24 and CID25 used for structure determinations with their complementary determining regions (CDRs) shown in red.

Movie S1. **Cholesterol binding sites in ACKR3.** ACKR3 was purified in the presence of cholesteryl hemisuccinate (CHS), resulting in occupation of multiple sites on the surface of the TM domain of ACKR3. Only the cholesterol moieties of CHS were well ordered (yellow ball and stick models). The structure of CID25-ACKR3·$CXCL12_{WT}$-CID24 (PDB entry 7SK3) shown in this movie has at least 8 ordered molecules of CHS. The complex is shown in various representations, with and without bound CXCL12 (green cartoon). Highlighted in the middle of a movie is the most commonly observed cholesterol binding site next to ACKR3-Trp169. Map density for each site is shown as a blue wire cage.

REFERENCES AND NOTES

1. P. M. Murphy, L. Heusinkveld, Multisystem multitasking by CXCL12 and its receptors CXCR4 and ACKR3. *Cytokine* **109**, 2–10 (2018).

2. K. Hattermann, R. Mentlein, An infernal trio: The chemokine CXCL12 and its receptors CXCR4 and CXCR7 in tumor biology. *Ann. Anat.* **195**, 103–110 (2013).

3. S. Rajagopal, J. Kim, S. Ahn, S. Craig, C. M. Lam, N. P. Gerard, C. Gerard, R. J. Lefkowitz, β-arrestin- but not G protein-mediated signaling by the “decoy” receptor CXCR7. *Proc. Natl. Acad. Sci. U.S.A.* **107**, 628–632 (2010).

4. A. Levoye, K. Balabanian, F. Baleux, F. Bachelerie, B. Lagane, CXCR7 heterodimerizes with CXCR4 and regulates CXCL12-mediated G protein signaling. *Blood* **113**, 6085–6093 (2009).

5. F. Saaber, D. Schütz, E. Miess, P. Abe, S. Desikan, P. Ashok Kumar, S. Balk, K. Huang, J. M. Beaulieu, S. Schulz, R. Stumm, ACKR3 regulation of neuronal migration requires ACKR3 phosphorylation, but not β-arrestin. *Cell Rep.* **26**, 1473–1488.e9 (2019).

6. G. Venkiteswaran, S. W. Lewellis, J. Wang, E. Reynolds, C. Nicholson, H. Knaut, Generation and dynamics of an endogenous, self-generated signaling gradient across a migrating tissue. *Cell* **155**, 674–687 (2013).

7. K. E. Luker, J. M. Steele, L. A. Mihalko, P. Ray, G. D. Luker, Constitutive and chemokine-dependent internalization and recycling of CXCR7 in breast cancer cells to degrade chemokine ligands. *Oncogene* **29**, 4599–4610 (2010).

8. A. Le Mercier, R. Bonnavion, W. Yu, M. W. Alnouri, S. Ramas, Y. Zhang, Y. Jäger, K. A. Roquid, H. W. Jeong, K. K. Sivaraj, H. Cho, X. Chen, B. Strilic, T. Sijmonsma, R. Adams, T. Schroeder, M. A. Rieger, S. Offermanns, GPR182 is an endothelium-specific atypical chemokine receptor that maintains hematopoietic stem cell homeostasis. *Proc. Natl. Acad. Sci. U.S.A.* **118**, e2021596118 (2021).

9. K. E. Quinn, D. I. Mackie, K. M. Caron, Emerging roles of atypical chemokine receptor 3 (ACKR3) in normal development and physiology. *Cytokine* **109**, 17–23 (2018).

10. M. Meyrath, M. Szpakowska, J. Zeiner, L. Massotte, M. P. Merz, T. Benkel, K. Simon, J. Ohnmacht, J. D. Turner, R. Krüger, V. Seutin, M. Ollert, E. Kostenis, A. Chevigné, The atypical chemokine receptor ACKR3/CXCR7 is a broad-spectrum scavenger for opioid peptides. *Nat. Commun.* **11**, 3033 (2020).

11. M. Szpakowska, A. M. Nevins, M. Meyrath, D. Rhainds, T. D'huys, F. Guité-Vinet, N. Dupuis, P. A. Gauthier, M. Counson, A. Kleist, G. St-Onge, J. Hanson, D. Schols, B. F. Volkman, N. Heveker, A. Chevigné, Different contributions of chemokine N-terminal features attest to a different ligand binding mode and a bias towards activation of ACKR3/CXCR7 compared with CXCR4 and CXCR3. *Br. J. Pharmacol.* **175**, 1419–1438 (2018).

12. M. S. Hanes, C. L. Salanga, A. B. Chowdry, I. Comerford, S. R. McColl, I. Kufareva, T. M. Handel, Dual targeting of the chemokine receptors CXCR4 and ACKR3 with novel engineered chemokines. *J. Biol. Chem.* **290**, 22385–22397 (2015).

13. X. Chen, P. Fan, M. M. Gleason, J. Jaen, L. Li, J. P. McMahon, J. Powers, Y. Zeng, P. Zhang, Modulators of CXCR7. WO2010/054006 A1 (2010).

14. M. Gustavsson, D. P. Dyer, C. Zhao, T. M. Handel, Kinetics of CXCL12 binding to atypical chemokine receptor 3 reveal a role for the receptor N terminus in chemokine binding. *Sci. Signal.* **12**, eaaw3657 (2019).

15. J. Ereño-Orbea, T. Sicard, H. Cui, J. Carson, P. Hermans, J. P. Julien, Structural basis of enhanced crystallizability induced by a molecular chaperone for antibody antigen-binding fragments. *J. Mol. Biol.* **430**, 322–336 (2018).

16. M. Szpakowska, M. Meyrath, N. Reynders, M. Counson, J. Hanson, J. Steyaert, A. Chevigné, Mutational analysis of the extracellular disulphide bridges of the atypical chemokine receptor ACKR3/CXCR7 uncovers multiple binding and activation modes for its chemokine and endogenous non-chemokine agonists. *Biochem. Pharmacol.* **153**, 299–309 (2018).

17. K. Liu, L. Wu, S. Yuan, M. Wu, Y. Xu, Q. Sun, S. Li, S. Zhao, T. Hua, Z. J. Liu, Structural basis of CXC chemokine receptor 2 activation and signalling. *Nature* **585**, 135–140 (2020).

18. M. A. Hanson, V. Cherezov, M. T. Griffith, C. B. Roth, V. P. Jaakola, E. Y. Chien, J. Velasquez, P. Kuhn, R. C. Stevens, A specific cholesterol binding site is established by the 2.8 A structure of the human β_2-adrenergic receptor. *Structure* **16**, 897–905 (2008).

19. D. H. Nguyen, D. Taub, Cholesterol is essential for macrophage inflammatory protein 1 β binding and conformational integrity of CC chemokine receptor 5. *Blood* **99**, 4298–4306 (2002).

20. D. H. Nguyen, D. Taub, CXCR4 function requires membrane cholesterol: Implications for HIV infection. *J. Immunol.* **168**, 4121–4126 (2002).

21. H. Zhang, K. Chen, Q. Tan, Q. Shao, S. Han, C. Zhang, C. Yi, X. Chu, Y. Zhu, Y. Xu, Q. Zhao, B. Wu, Structural basis for chemokine recognition and receptor activation of chemokine receptor CCR5. *Nat. Commun.* **12**, 4151 (2021).

22. T. Ngo, B. S. Stephens, M. Gustavsson, L. G. Holden, R. Abagyan, T. M. Handel, I. Kufareva, Crosslinking-guided geometry of a complete CXC receptor-chemokine complex and the basis of chemokine subfamily selectivity. *PLOS Biol.* **18**, e3000656 (2020).

23. L. J. Drury, J. J. Ziarek, S. Gravel, C. T. Veldkamp, T. Takekoshi, S. T. Hwang, N. Heveker, B. F. Volkman, M. B. Dwinell, Monomeric and dimeric CXCL12 inhibit metastasis through distinct CXCR4 interactions and signaling pathways. *Proc. Natl. Acad. Sci. U.S.A.* **108**, 17655–17660 (2011).

24. M. Gustavsson, L. Wang, N. van Gils, B. S. Stephens, P. Zhang, T. J. Schall, S. Yang, R. Abagyan, M. R. Chance, I. Kufareva, T. M. Handel, Structural basis of ligand interaction with atypical chemokine receptor 3. *Nat. Commun.* **8**, 14135 (2017).

25. B. Benredjem, M. Girard, D. Rhainds, G. St-Onge, N. Heveker, Mutational analysis of atypical chemokine receptor 3 (ACKR3/CXCR7) interaction with its chemokine ligands CXCL11 and CXCL12. *J. Biol. Chem.* **292**, 31–42 (2017).

26. A. Jaracz-Ros, G. Bernadat, P. Cutolo, C. Gallego, M. Gustavsson, E. Cecon, F. Baleux, I. Kufareva, T. M. Handel, F. Bachelerie, A. Levoye, Differential activity and selectivity of N-terminal modified CXCL12 chemokines at the CXCR4 and ACKR3 receptors. *J. Leukoc. Biol.* **107**, 1123–1135 (2020).

27. M. Canals, D. J. Scholten, S. de Munnik, M. K. Han, M. J. Smit, R. Leurs, Ubiquitination of CXCR7 controls receptor trafficking. *PLOS ONE* **7**, e34192 (2012).

28. B. T. DeVree, J. P. Mahoney, G. A. Vélez-Ruiz, S. G. Rasmussen, A. J. Kuszak, E. Edwald, J. J. Fung, A. Manglik, M. Masureel, Y. Du, R. A. Matt, E. Pardon, J. Steyaert, B. K. Kobilka, R. K. Sunahara, Allosteric coupling from G protein to the agonist-binding pocket in GPCRs. *Nature* **535**, 182–186 (2016).

29. N. R. Latorraca, A. J. Venkatakrishnan, R. O. Dror, GPCR dynamics: Structures in motion. *Chem. Rev.* **117**, 139–155 (2017).

30. W. I. Weis, B. K. Kobilka, The molecular basis of G protein-coupled receptor activation. *Annu. Rev. Biochem.* **87**, 897–919 (2018).

31. J. A. Goncalves, K. South, S. Ahuja, E. Zaitseva, C. A. Opefi, M. Eilers, R. Vogel, P. J. Reeves, S. O. Smith, Highly conserved tyrosine stabilizes the active state of rhodopsin. *Proc. Natl. Acad. Sci. U.S.A.* **107**, 19861–19866 (2010).

32. S. O. Smith, Deconstructing the transmembrane core of class A G protein-coupled receptors. *Trends Biochem. Sci.* **46**, 1017–1029 (2021).

33. A. Fumagalli, J. Heuninck, A. Pizzoccaro, E. Moutin, J. Koenen, M. Séveno, T. Durroux, M. P. Junier, G. Schlecht-Louf, F. Bachelerie, D. Schütz, R. Stumm, M. J. Smit, N. C. Guérineau, S. Chaumont-Dubel, P. Marin, The atypical chemokine receptor 3 interacts with Connexin 43 inhibiting astrocytic gap junctional intercellular communication. *Nat. Commun.* **11**, 4855 (2020).

34. C. Koch, J. Engele, Functions of the CXCL12 receptor ACKR3/CXCR7-what has been perceived and what has been overlooked. *Mol. Pharmacol.* **98**, 577–585 (2020).

35. X. Han, S. D. Tachado, H. Koziel, W. A. Boisvert, Leu128(3.43) (l128) and Val247(6.40) (V247) of CXCR1 are critical amino acid residues for G protein coupling and receptor activation. *PLOS ONE* **7**, e42765 (2012).

36. X. Deupi, P. Edwards, A. Singhal, B. Nickle, D. Oprian, G. Schertler, J. Standfuss, Stabilized G protein binding site in the structure of constitutively active metarhodopsin-II. *Proc. Natl. Acad. Sci. U.S.A.* **109**, 119–124 (2012).

37. N. Montpas, G. St-Onge, N. Nama, D. Rhainds, B. Benredjem, M. Girard, G. Hickson, V. Pons, N. Heveker, Ligand-specific conformational transitions and intracellular transport are required for atypical chemokine receptor 3-mediated chemokine scavenging. *J. Biol. Chem.* **293**, 893–905 (2018).

38. J. Jumper, R. Evans, A. Pritzel, T. Green, M. Figurnov, O. Ronneberger, K. Tunyasuvunakool, R. Bates, A. Žídek, A. Potapenko, A. Bridgland, C. Meyer, S. A. A. Kohl, A. J. Ballard, A. Cowie, B. Romera-Paredes, S. Nikolov, R. Jain, J. Adler, T. Back, S. Petersen, D. Reiman, E. Clancy, M. Zielinski, M. Steinegger, M. Pacholska, T. Berghammer, S. Bodenstein, D. Silver, O. Vinyals, A. W. Senior, K. Kavukcuoglu, P. Kohli, D. Hassabis, Highly accurate protein structure prediction with AlphaFold. *Nature* **596**, 583–589 (2021).

39. D. Maussang, A. Mujić-Delić, F. J. Descamps, C. Stortelers, P. Vanlandschoot, M. Stigter-van Walsum, H. F. Vischer, M. van Roy, M. Vosjan, M. Gonzalez-Pajuelo, G. A. van Dongen, P. Merchiers, P. van Rompaey, M. J. Smit, Llama-derived single variable domains (nanobodies) directed against chemokine receptor CXCR7 reduce head and neck cancer cell growth in vivo. *J. Biol. Chem.* **288**, 29562–29572 (2013).

40. S. Richard-Bildstein, H. Aissaoui, J. Pothier, G. Schäfer, C. Gnerre, E. Lindenberg, F. Lehembre, L. Pouzol, P. Guerry, Discovery of the potent, selective, orally available CXCR7 antagonist ACT-1004–1239. *J. Med. Chem.* **63**, 15864–15882 (2020).

41. M. P. Crump, J. H. Gong, P. Loetscher, K. Rajarathnam, A. Amara, F. Arenzana-Seisdedos, J. L. Virelizier, M. Baggiolini, B. D. Sykes, I. Clark-Lewis, Solution structure and basis for

functional activity of stromal cell-derived factor-1; Dissociation of CXCR4 activation from binding and inhibition of HIV-1. *EMBO J.* **16**, 6996–7007 (1997).

42. B. S. Stephens, T. Ngo, I. Kufareva, T. M. Handel, Functional anatomy of the full-length CXCR4-CXCL12 complex systematically dissected by quantitative model-guided mutagenesis. *Sci. Signal.* **13**, eaay5024 (2020).

43. T. F. Miles, K. Spiess, K. M. Jude, N. Tsutsumi, J. S. Burg, J. R. Ingram, D. Waghray, G. M. Hjorto, O. Larsen, H. L. Ploegh, M. M. Rosenkilde, K. C. Garcia, Viral GPCR US28 can signal in response to chemokine agonists of nearly unlimited structural degeneracy. *eLife* **7**, e35850 (2018).

44. L. M. Wingler, R. J. Lefkowitz, Conformational basis of G protein-coupled receptor signaling versatility. *Trends Cell Biol.* **30**, 736–747 (2020).

45. D. Ahn, D. Ham, K. Y. Chung, The conformational transition during G protein-coupled receptor (GPCR) and G protein interaction. *Curr. Opin. Struct. Biol.* **69**, 117–123 (2021).

46. Q. Chen, M. Plasencia, Z. Li, S. Mukherjee, D. Patra, C. L. Chen, T. Klose, X. Q. Yao, A. A. Kossiakoff, L. Chang, P. C. Andrews, J. J. G. Tesmer, Structures of rhodopsin in complex with G-protein-coupled receptor kinase 1. *Nature* **595**, 600–605 (2021).

47. L. M. Wingler, M. Elgeti, D. Hilger, N. R. Latorraca, M. T. Lerch, D. P. Staus, R. O. Dror, B. K. Kobilka, W. L. Hubbell, R. J. Lefkowitz, Angiotensin analogs with divergent bias stabilize distinct receptor conformations. *Cell* **176**, 468–478.e11 (2019).

48. C. M. Suomivuori, N. R. Latorraca, L. M. Wingler, S. Eismann, M. C. King, A. L. W. Kleinhenz, M. A. Skiba, D. P. Staus, A. C. Kruse, R. J. Lefkowitz, R. O. Dror, Molecular mechanism of biased signaling in a prototypical G protein-coupled receptor. *Science* **367**, 881–887 (2020).

49. V. Odemis, J. Lipfert, R. Kraft, P. Hajek, G. Abraham, K. Hattermann, R. Mentlein, J. Engele, The presumed atypical chemokine receptor CXCR7 signals through Gi/o proteins in primary rodent astrocytes and human glioma cells. *Glia* **60**, 372–381 (2012).

50. M. Gustavsson, Y. Zheng, T. M. Handel, Production of chemokine/chemokine receptor complexes for structural biophysical studies. *Methods Enzymol.* **570**, 233–260 (2016).

51. T. K. Ritchie, Y. V. Grinkova, T. H. Bayburt, I. G. Denisov, J. K. Zolnerciks, W. M. Atkins, S. G. Sligar, Chapter 11—Reconstitution of membrane proteins in phospholipid bilayer nanodiscs. *Methods Enzymol.* **464**, 211–231 (2009).

52. P. K. Dominik, M. T. Borowska, O. Dalmas, S. S. Kim, E. Perozo, R. J. Keenan, A. A. Kossiakoff, Conformational chaperones for structural studies of membrane proteins using antibody phage display with nanodiscs. *Structure* **24**, 300–309 (2016).

53. K. R. Miller, A. Koide, B. Leung, J. Fitzsimmons, B. Yoder, H. Yuan, M. Jay, S. S. Sidhu, S. Koide, E. J. Collins, T cell receptor-like recognition of tumor in vivo by synthetic antibody fragment. *PLOS ONE* **7**, e43746 (2012).

54. P. K. Dominik, A. A. Kossiakoff, Phage display selections for affinity reagents to membrane proteins in nanodiscs. *Methods Enzymol.* **557**, 219–245 (2015).

55. M. T. Borowska, P. K. Dominik, S. A. Anghel, A. A. Kossiakoff, R. J. Keenan, A YidC-like protein in the archaeal plasma membrane. *Structure* **23**, 1715–1724 (2015).

56. A. I. Alexandrov, M. Mileni, E. Y. Chien, M. A. Hanson, R. C. Stevens, Microscale fluorescent thermal stability assay for membrane proteins. *Structure* **16**, 351–359 (2008).

57. X. Chen, D. R. Dragoli, P. Fan, M. M. Gleason, J. C. Jaen, L. Li, J. P. McMahon, J. Powers, Y. Zeng, P. Zhang, J. Fan, Modulators of CXCR7. U.S. Patent US8853202B2 (2010).

58. A. Punjani, J. L. Rubinstein, D. J. Fleet, M. A. Brubaker, cryoSPARC: Algorithms for rapid unsupervised cryo-EM structure determination. *Nat. Methods* **14**, 290–296 (2017).

59. A. Punjani, H. Zhang, D. J. Fleet, Non-uniform refinement: Adaptive regularization improves single-particle cryo-EM reconstruction. *Nat. Methods* **17**, 1214–1221 (2020).

60. J. Zivanov, T. Nakane, B. O. Forsberg, D. Kimanius, W. J. Hagen, E. Lindahl, S. H. Scheres, New tools for automated high-resolution cryo-EM structure determination in RELION-3. *eLife* **7**, e42166 (2018).

61. P. D. Adams, P. V. Afonine, G. Bunkóczi, V. B. Chen, I. W. Davis, N. Echols, J. J. Headd, L. W. Hung, G. J. Kapral, R. W. Grosse-Kunstleve, A. J. McCoy, N. W. Moriarty, R. Oeffner, R. J. Read, D. C. Richardson, J. S. Richardson, T. C. Terwilliger, P. H. Zwart, PHENIX: A comprehensive Python-based system for macromolecular structure solution. *Acta Crystallogr. D Biol. Crystallogr.* **66**, 213–221 (2010).

62. L. G. Trabuco, E. Villa, K. Mitra, J. Frank, K. Schulten, Flexible fitting of atomic structures into electron microscopy maps using molecular dynamics. *Structure* **16**, 673–683 (2008).

63. W. Humphrey, A. Dalke, K. Schulten, VMD: Visual molecular dynamics. *J. Mol. Graph.* **14**, 27–38 (1996).

64. Y. Namkung, C. Le Gouill, V. Lukashova, H. Kobayashi, M. Hogue, E. Khoury, M. Song, M. Bouvier, S. A. Laporte, Monitoring G protein-coupled receptor and β-arrestin trafficking in live cells using enhanced bystander BRET. *Nat. Commun.* **7**, 12178 (2016).

65. I. Masuho, K. A. Martemyanov, N. A. Lambert, Monitoring G protein activation in cells with BRET. *Methods Mol. Biol.* **1335**, 107–113 (2015).

66. M. Wijtmans, D. Maussang, F. Sirci, D. J. Scholten, M. Canals, A. Mujić-Delić, M. Chong, K. L. Chatalic, H. Custers, E. Janssen, C. de Graaf, M. J. Smit, I. J. de Esch, R. Leurs, Synthesis, modeling and functional activity of substituted styrene-amides as small-molecule CXCR7 agonists. *Eur. J. Med. Chem.* **51**, 184–192 (2012).

67. S. Udenfriend, L. D. Gerber, L. Brink, S. Spector, Scintillation proximity radioimmunoassay utilizing 125I-labeled ligands. *Proc. Natl. Acad. Sci. U.S.A.* **82**, 8672–8676 (1985).
